# Machine-Learning-Based Prediction of Plant Cuticle–Air Partition Coefficients for Organic Pollutants: Revealing Mechanisms from a Molecular Structure Perspective

**DOI:** 10.3390/molecules29061381

**Published:** 2024-03-20

**Authors:** Tianyun Tao, Cuicui Tao, Tengyi Zhu

**Affiliations:** 1 College of Agriculture, Yangzhou University, Yangzhou 225009, China; 2 School of Environmental Science and Engineering, Yangzhou University, Yangzhou 225127, China

**Keywords:** organic pollutants, plant cuticle–air partition coefficient, QSPR, machine learning

## Abstract

Accurately predicting plant cuticle–air partition coefficients (*K*_ca_) is essential for assessing the ecological risk of organic pollutants and elucidating their partitioning mechanisms. The current work collected 255 measured *K*_ca_ values from 25 plant species and 106 compounds (dataset (I)) and averaged them to establish a dataset (dataset (II)) containing *K*_ca_ values for 106 compounds. Machine-learning algorithms (multiple linear regression (MLR), multi-layer perceptron (MLP), k-nearest neighbors (KNN), and gradient-boosting decision tree (GBDT)) were applied to develop eight QSPR models for predicting *K*_ca_. The results showed that the developed models had a high goodness of fit, as well as good robustness and predictive performance. The GBDT-2 model ($R_{\mathrm{adj}}^{2}$ = 0.925, $Q_{\mathrm{LOO}}^{2}$ = 0.756, $Q_{\mathrm{BOOT}}^{2}$ = 0.864, $R_{\mathrm{ext}}^{2}$ = 0.837, $Q_{\mathrm{ext}}^{2}$ = 0.811, and *CCC* = 0.891) is recommended as the best model for predicting *K*_ca_ due to its superior performance. Moreover, interpreting the GBDT-1 and GBDT-2 models based on the Shapley additive explanations (SHAP) method elucidated how molecular properties, such as molecular size, polarizability, and molecular complexity, affected the capacity of plant cuticles to adsorb organic pollutants in the air. The satisfactory performance of the developed models suggests that they have the potential for extensive applications in guiding the environmental fate of organic pollutants and promoting the progress of eco-friendly and sustainable chemical engineering.

## 1. Introduction

Natural and human activities release massive quantities of organic pollutants into the atmosphere [1]. These pollutants are intercepted by terrestrial vegetation and transferred to terrestrial ecosystems, where they accumulate through the food chain to higher nutrient levels [2,3]. The plant cuticle acts as the main interface for the exchange of organic pollutants between the air phase and the plant. It also serves as the main barrier to the interception of atmospheric pollutants [4]. Apart from the uptake and release of organic pollutants, the plant cuticle is also recognized as an accumulation chamber for organic pollutants [5,6]. The partitioning of organic compounds between the cuticle and air is of great interest due to airborne organic pollutants’ strong affinity to plant cuticles and their potential toxicity [7].

The exchange of organic contaminants between plant cuticles and air is typically evaluated through the plant cuticle–air partition coefficient (*K*_ca_) [8,9]. The partition coefficient, *K*_ca_, is generally determined by the ratio of the equilibrium concentration of organic contaminants between the isolated cuticle membranes (CMs) or polymer matrix membranes (MX) and the air, indicating the plant’s capacity to uptake airborne organic pollutants [10,11]. It is important to note that, at present, there is a paucity of experimentally measured *K*_ca_ values, and the interaction mechanisms of organic pollutants between plant cuticles and air are still obscure [12]. To the best of our knowledge, only a few hundred *K*_ca_ values for organic pollutants between a few plants and air have been measured experimentally [6]. The scarcity of these experimental *K*_ca_ values is mainly attributed to the laborious and time-consuming nature of conducting experiments to measure them, as well as the challenges posed by the diversity of plant species and types of organic contaminants [13,14]. This has also become an insurmountable bottleneck in assessing the ecological risk of organic pollutants by understanding their accumulation in plants and the exchange between the atmosphere and plants. Therefore, it could be valuable to focus on a straightforward and efficient method for predicting the *K*_ca_ values of organic pollutants to assess air quality and associated ecological risks.

Developing models with the ability to capture the molecular interactions between different compounds and plant cuticles is a complex task. When few independent variables are included, it is difficult, or sometimes even impossible, for a model to adequately reflect the mechanisms of interest precisely due to the limited number of independent variables available, which are representative of the influencing factors [2,15]. Some studies have solved this problem by developing poly-parameter linear free-energy relationship (pp-LFER) models [6,10]. However, the application of these models has been limited by the scarcity of Abrahamic descriptors and the fact that Abrahamic descriptors are not applicable to certain non-polar compounds [16]. This hinders the development and application of mechanism-based predictive models.

The relationship between the physical and chemical properties of the compound and the descriptors that quantify the molecular structural properties of compounds, namely the quantitative structural property relationship, provides a potential shortcut for clarifying the interaction between molecules and plant cuticles [17,18]. Currently, only one study has demonstrated the potential of QSPR models by developing a QSPR model for predicting *K*_ca_ [2]. However, the dataset used in this study contained only 49 log *K*_ca_ values, and these 49 organic compounds were measured with the same plant cuticle. The limited amount of available data poses a challenge to the development of a machine-learning model with a wide range of applications for assessing the ecotoxicity of compounds in various plant species.

Moreover, the challenge lies in capturing the complex relationship between *K*_ca_ and molecular structural properties from the increased amount of data and independent variables. Although the multiple linear regression (MLR) algorithm is convenient, efficient, and transparent in modeling, it is not applicable to complex nonlinear relationships, resulting in poor model performance [19]. We believe that the nonlinear machine-learning algorithm is the best candidate for *K*_ca_ prediction because of its powerful nonlinear processing capability [20,21]. For example, a study developed a QSPR model using the multi-layer perceptron (MLP) algorithm to predict the acute oral toxicity of pesticides in rats, achieving a high accuracy of 0.963 [22]. However, these types of models often have a “black box” nature [23]. Uncovering the dominant factors affecting the partitioning behavior of organic pollutants between plant cuticles and air from data and revealing their implicit effects while ensuring the predictability of the models is still a great challenge.

This study aimed to develop QSPR models by establishing relationships between measured *K*_ca_ values and molecular descriptors corresponding to organic pollutants in existing studies in order to ascertain the main mechanisms underlying the partitioning behavior of pollutants between the plant cuticle and the air. To achieve this, a comprehensive dataset of 255 *K*_ca_ values for 106 compounds and 25 plant species was collected to broaden the application domain of the models. In addition to the traditional linear algorithm (MLR), three popular nonlinear algorithms, namely MLP, k-nearest neighbors (KNN), and gradient-boosting decision tree (GBDT), were employed to develop QSPR models. Following the OECD guidelines [24], the models built using different machine-learning algorithms were rigorously and comprehensively evaluated to determine which of the QSPR models predicted *K*_ca_ most accurately. The application domains of the models were limited using the leverage method. Finally, the mechanism underlying plant cuticles’ adsorption of airborne organic pollutants was elucidated through the help of Shapley additive explanations (SHAP), thereby extracting some tacit or novel knowledge about the interaction between plant cuticles and organic pollutants. It is worth mentioning that the models developed in this study can estimate the accumulation of organics at the plant cuticle–air interface accurately even in the absence of experimental data, and they can provide valuable environmental information to guide the risk assessment and regulation of organic pollutants.

## 2. Results and Discussion

### 2.1. Development of the QSPR Model

**Figure 1:**
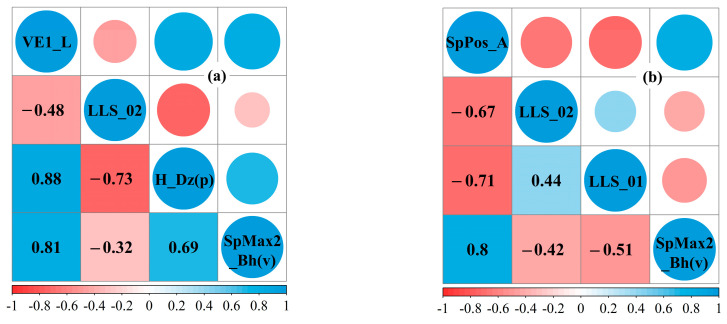
Descriptor correlation graph of the QSPR models. (**a**) Datasets (I); (**b**) Datasets (II).

The MLR algorithm selected the most appropriate descriptors from the pool of descriptors obtained from our initial screening and developed the QSPR models. Although the overall performance of the models improved as the number of descriptors increased (Figure S1), after the number of descriptors reached 5, descriptors with a *VIF* value greater than 10 were present in both datasets. Thus, the number of optimal descriptor combinations for both datasets was determined to be four after removing as much redundant information as possible for the QSPR models [25]. From Figure 1, it can be observed that the descriptors are not excessively correlated with each other. The selected best descriptors and their associated statistical indicators are presented in Table 1. The *p*-values of these eight descriptors are <0.5, indicating that they are statistically significant. The linear QSPR models developed with the selected descriptors were as follows:

**MLR-1** (dataset (I)):

log *K*_ca_ = 2.006 *VE1_L* + 14.945 *LLS_02* + 0.094 *H_Dz(p)* − 1.044 *SpMax2_Bh(v)* − 13.433 (1)

*n*_tra_ = 204, $R_{\mathrm{adj}}^{2}$ = 0.873, $Q_{\mathrm{LOO}}^{2}$ = 0.869, $Q_{\mathrm{BOOT}}^{2}$ = 0.872, *RMSE*_tra_ = 1.101;
*n*_ext_ = 51, $R_{\mathrm{ext}}^{2}$ = 0.839, $Q_{\mathrm{ext}}^{2}$ = 0.835, *RMSE*_ext_ = 1.296.

**MLR-2** (dataset (II)):

log *K*_ca_ = 1.325 *SpPos_A* + 12.317 *LLS_02* + 7.385 *LLS_01* − 1.517 *SpMax2_Bh(v)* − 15.724 (2)

*n*_tra_ = 84, $R_{\mathrm{adj}}^{2}$ = 0.891, $Q_{\mathrm{LOO}}^{2}$ = 0.874, $Q_{\mathrm{BOOT}}^{2}$ = 0.886, *RMSE*_tra_ = 0.987;
*n*_ext_ = 22, $R_{\mathrm{ext}}^{2}$ = 0.833, $Q_{\mathrm{ext}}^{2}$ = 0.807, *RMSE*_ext_ = 1.466.

Three nonlinear algorithms, namely MLP, KNN, and GBDT, were employed to explore the nonlinear relationship between the *K*_ca_ of organic pollutants and their molecular structures, with a view to developing more accurate models. For the same dataset, the nonlinear models were trained using the same molecular descriptors as the linear models. Eventually, six nonlinear QSPR models were developed based on two datasets and three machine-learning algorithms. The results of the hyperparameter search for the models are presented in Table S1. The measured and predicted log *K*_ca_ values for the MLR-1 model, MLP-1 model, KNN-1 model, and GBDT-1 model are provided in Table S2, while the values associated with the models developed based on dataset (II) are presented in Table S3. The most stringent model validation criteria in the field of QSPR research were employed in this study to assess the performance of models, including $R_{\mathrm{adj}}^{2}$ > 0.7, $Q_{\mathrm{LOO}}^{2}$ > 0.6, $Q_{\mathrm{BOOT}}^{2}$ > 0.6, $R_{\mathrm{ext}}^{2}$ > 0.7, $Q_{\mathrm{ext}}^{2}$ > 0.6, *CCC* > 0.85, and minimize error values [26,27]. The values of the validation parameters for the eight QSPR models are shown in Table 2.

**Figure 2:**
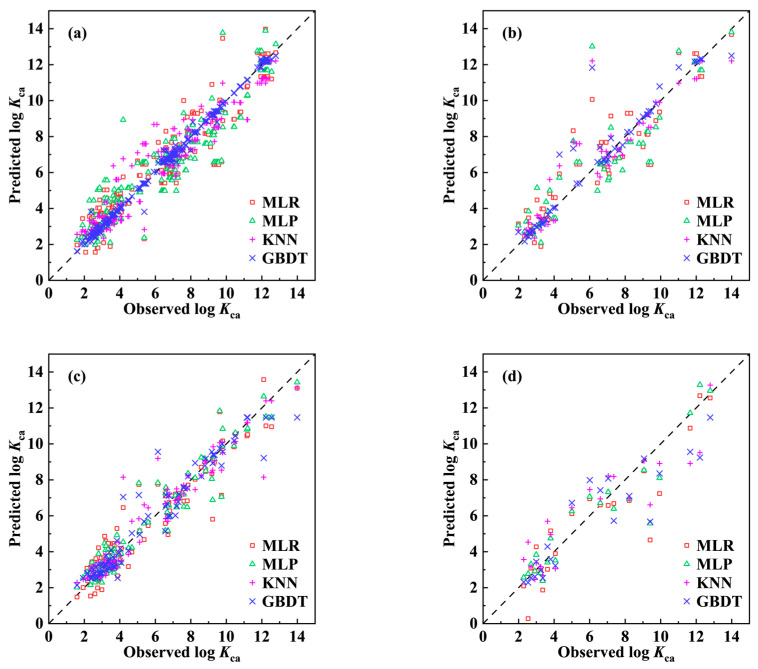
Plots of the observed versus predicted for log *K*_ca_ based on dataset (I). (**a**) Training set; (**b**) Test set; Plots of the observed versus predicted for log *K*_ca_ based on dataset (II). (**c**) Training set; (**d**) Test set.

As shown in Table 2, the $R_{\mathrm{adj}}^{2}$ (0.850–0.995) of the four models (MLR-1, MLP-1, KNN-1, and GBDT-1 model) developed based on dataset (I) exceeded the standard thresholds, demonstrating excellent goodness of fit. The stability parameters $Q_{\mathrm{LOO}}^{2}$ and $Q_{\mathrm{BOOT}}^{2}$ had values of 0.678–0.936 and 0.676–0.964, respectively, above the acceptable thresholds, indicating that the models are statistically robust and have fair internal accuracy [28]. In terms of external predictive power, the $R_{\mathrm{ext}}^{2}$ (0.790–0.911), $Q_{\mathrm{ext}}^{2}$ (0.784–0.902), and *CCC* (0.889–0.952) of these models were much greater than the strict standard thresholds, implying that all four models achieved fairly reasonable predictions [29]. The measured and predicted log *K*_ca_ values for the training and test sets are plotted in Figure 2a and Figure 2b, respectively. For the training and test sets of each model, the data points followed a similar discrete pattern, with all of them being close to the 1:1 line, proving that these four models had high-level accuracy and prediction abilities [30,31]. The consistency of the training and test set errors indicated that these models had similar internal and external prediction accuracies, confirming the excellent external prediction abilities of these models [30]. Overall, both the linear and nonlinear QSPR models developed based on dataset (I) were acceptable.

Multiple verification parameters were also calculated to evaluate the performance of the models (MLR-2, MLP-2, KNN-2, and GBDT-2 model) developed based on dataset (II) (Table 2). The internal validation results derived from the use of the data points in the training set were $R_{\mathrm{adj}}^{2}$ = 0.891–0.925, $Q_{\mathrm{LOO}}^{2}$ = 0.661–0.874, and $Q_{\mathrm{BOOT}}^{2}$ = 0.802–0.886, indicating that the models exhibited excellent internal predictability and stability. The external validation results derived from the use of the data points in the test set were $R_{\mathrm{ext}}^{2}$ = 0.821–0.887, $Q_{\mathrm{ext}}^{2}$ = 0.807–0.884, and *CCC* = 0.891–0.940, demonstrating the superior performance of the models in predicting external data. In addition, the error-based statistical metrics further demonstrated the “good” quality of these models in predicting log *K*_ca_ values for the training set and test set. Scatter plots of the log *K*_ca_ values measured and predicted by the four models developed using dataset (II) are shown in Figure 2c,d. The data points were more concentrated on the 1:1 line than those in dataset (I), indicating that the four models also have the appropriate ability to predict log *K*_ca_ values. The results of our rigorous validation testing indicated that these four models performed satisfactorily in various aspects.

### 2.2. Applicability Domain

**Figure 3:**
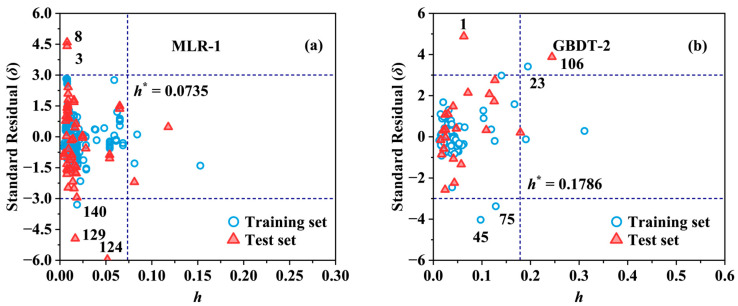
Application domain characterized by Williams plots: the MLR-1 (**a**) and GBDT-2 (**b**) models for log *K*_ca_.

The Williams plots shown in Figure 3 and Figure S2 established the structure range of the compounds for which the model could reliably predict log *K*_ca_ values. Although structural outliers were found in all eight models, most of them fell into the category of “good high leverage” points with low *δ* (|*δ|* ≤ 3). These points were predicted with high accuracy, ensuring the stability and generalization performance of the models and extending their applicability domains to some extent [32,33]. The MLP-1, KNN-2, and GBDT-2 models contained one, two, and two structural outliers with |*δ*| > 3 as well (Figure S2a,f and Figure 3b), respectively. This may be caused by the unique structure of these three compounds and the limited structural representation of the selected descriptors [34]. Information related to the response outliers in datasets (I) and (II) is listed in Tables S4 and S5. Decachlorobiphenyl (ID: 124) and Tetrachlorobiphenyl (ID: 129) in dataset (I) were detected as response outliers in all of the four models developed by the four algorithms. This inaccurate prediction might have been caused by the fact that the molecular descriptors did not capture information about the key effects of plant cuticle adsorption on these two compounds [33]. In addition, the variability of the experimental values may also have an impact on the model predictions [35]. For example, the *K*_ca_ value for Hexachlorobenzene (ID: 74) was probably underestimated excessively by Gobas, McNeil, Lovett-Doust and Haffner [36], and, therefore, was not in the AD range of the KNN-1 model and the GBDT-1 model when compared to the value measured by Sabljic, Guesten, Schoenherr and Riederer [5] (log *K*_ca_ = 4.30 versus 6.78~7.28). By taking the mean value instead, the models developed based on dataset (II) predicted the *K*_ca_ value for Hexachlorobenzene (ID: 24) with good accuracy. Overall, the presence of most data points in the applicability domain demonstrated the validity and good performance of the models [37].

### 2.3. Mechanism Interpretation

**Figure 4:**
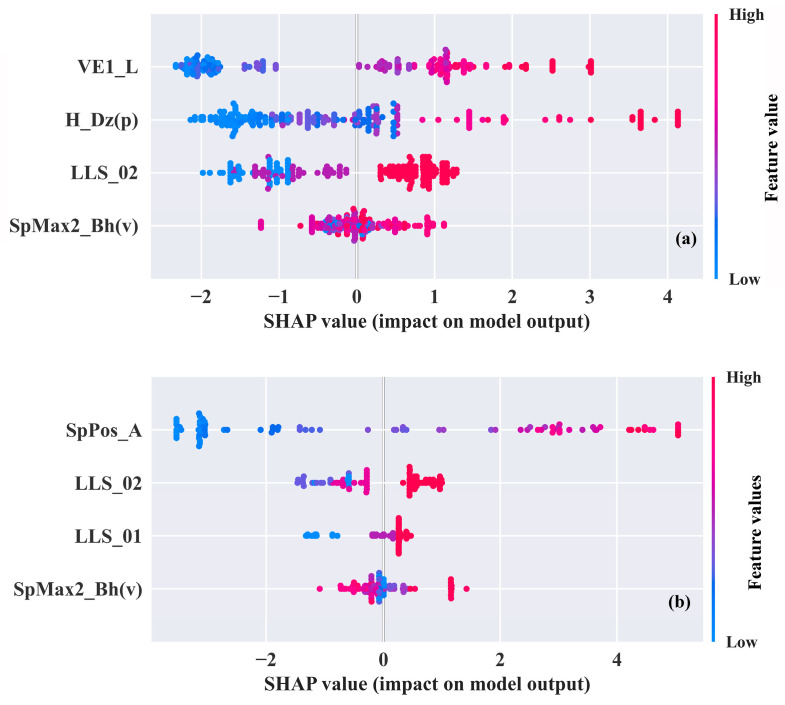
Relationship between SHAP value and the values of different descriptors for dataset (I) (**a**) and dataset (II) (**b**).

In addition to assessing the performance of each model, SHAP values were analyzed to interpret both the GBDT-1 model and the GBDT-2 model and determine whether the models were consistent with known mechanisms [38]. The SHAP summary plot shown in Figure 4 succinctly and clearly shows the relationship between the SHAP values and each descriptor, making it easy to discern whether there is a positive or negative correlation between the descriptor and log *K*_ca_. For example, compounds with a large *VE1_L* (red color) typically have higher SHAP values, indicating higher log *K*_ca_ values. Thus, the correlation between *VE1_L* and log *K*_ca_ can be considered positive. The descriptors are sorted on the *y*-axis in descending order by the mean of the absolute value of SHAP, i.e., the descriptor contribution.

Figure 4 shows that the contributions of *LLS_02* and *SpMax2_Bh(v)* to log *K*_ca_ are consistently in the top four positions in both the GBDT-1 model and GBDT-2 model, suggesting that these two descriptors have a dominant effect on the plant cuticle’s capacity to adsorb organic pollutants. The descriptor *LLS_02* is a lead-like score modified by Monge, Arrault, Marot and Morin-Allory [39], and it was used to screen compounds that qualify as leads in drug discovery [40]. The score was determined based on eight rules [39]: compounds that met all the rules had an *LLS_02* value equal to 1, and the more rules that were violated, the lower the *LLS_02* value [41]. From these eight rules, it was found that compounds with lower *LLS_02* values had high molecular weights, as well as a high number of hydrogen bond donors and hydrogen bond acceptors. High-molecular-weight compounds are usually difficult to pass through phospholipid membranes, and increasing the number of hydrogen bond donors and hydrogen bond acceptors makes these compounds more hydrophilic [42]. Therefore, a decrease in log *K*_ca_ could be expected for compounds with lower *LLS_02* values. The descriptor *SpMax2_Bh(v)* was largest eigenvalue n. 2 of Burden matrix weighted by van der Waals volume, and belonged to Burden eigenvalues [43]. Burden eigenvalues were calculated from the Burden matrix *Bh(w)*, and the diagonal elements of the adjacency matrix are van der Waals volume. These values were related to molecular branching, the presence of heteroatoms, and bond multiplicity [44,45]. The van der Waals volume contributed to the lipophilicity of the molecule, and the increase in the *SpMax2_Bh(v)* value logically should have led to an increase in the *K*_ca_ value [46]. However, as presented in Figure 4a,b, how the *SpMax2_Bh(v)* values regulated the partitioning of organic pollutants between plant cuticles and the air was complicated. This might be due to the limited number of data points making it difficult for the models to adequately respond to the mechanisms involved. Similar to *LLS_02*, *LLS_01* is a lead-like score. The score was determined by six rules (molecular weight, hydrogen bond donors, hydrogen bond acceptors, number of rotatable bonds, etc.) [47]. Therefore, there was also a positive correlation between *LLS_01* and log *K*_ca_.

The *VE1_L*, *H_Dz(p)*, and *SpPos_A* belonged to the category of 2D matrix-based descriptors. The *VE1_L* was based on the Laplace matrix, which provides the number of spanning trees for molecular graphing. This quantity reflected the structural complexity of polycyclic molecules, with higher spanning-tree quantities indicating greater molecular structural complexity [48]. Generally, compounds with polycyclic structures were more stable and conducive to the adsorption of compounds by the plant cuticle, meaning that they should have shown increased *K*_ca_ values [29,49]. The descriptor *H_Dz(p)* was based on the Barysz matrix, weighted by the polarizability [50]. The Barysz matrix was considered to be related to the presence of heteroatoms and multiple bonds in molecules [48]. The magnitude of the polarizability was influenced by molecular size, structure, and electron distribution [51]. The greater the polarization rate, the stronger the polarity of the molecules, and the stronger the intermolecular interactions [52]. Molecules tended to distribute in the plant cuticle through intermolecular forces, leading to an increase in *K*_ca_ values [53]. The *SpPos_A* was calculated by summing the positive eigenvalues from the adjacency matrix, encoding information about molecular size, molecular branching, and molecular complexity [54,55,56]. Figure 4 indicates that log *K*_ca_ is proportional to *SpPos_A*.

According to the results of this study, molecular weight, molecular size, molecular branching, molecular complexity, the number of hydrogen bond donors, the number of hydrogen bond acceptors, the presence of heteroatoms, bond multiplicity, polycyclic structure, and polarizability are the main molecular structure features that affect the capacity of plant cuticles to adsorb airborne organic pollutants.

### 2.4. Model Comparison

To determine which model was most effective at predicting *K*_ca_ values, cumulative distribution plots of the residuals (Figure S3) were plotted to depict the predictive effectiveness of the eight models. As shown in Figure S3, if the residuals fall in the −1 to 1 interval with a higher percentage, the model predicts the log *K*_ca_ values more accurately. Our comparison among the four models developed based on dataset (I) indicated that the GBDT-1 model was significantly better than the other three models, while the GBDT-2 model was found to show the best performance among the four models developed based on dataset (II). The superiority of the GBDT-1 and GBDT-2 models in predicting the log *K*_ca_ values can also clearly be observed in the scatter plots of the real predicted values (Figure 2). Although the GBDT-1 model performed better than the GBDT-2 model in terms of the validation parameters (Table 2), the repetitive data points in dataset (I) exacerbated the risk of data leakage. Therefore, the GBDT-2 model is recommended as a useful tool for predicting the *K*_ca_ values of organic pollutants.

To further evaluate the eight QSPR models, several existing models for predicting log *K*_ca_ values were collected and compared. The differences in datasets, descriptors, and validation metrics between the different studies reporting these models increased the difficulty of the comparison. The details of the comparison are presented in Table S6. The number of compounds simulated in the early studies was not more than a hundred; the types of compounds were concentrated, and the distribution behavior was more regular, resulting in a high fitting accuracy for their models [2,10]. Eddula, Xu, Jiang, Huang, Tirumala, Liu, Acree and Abraham [6] collected 215 measured *K*_ca_ values and established pp-LFER models with excellent accuracy. However, the application of these models was limited by the number of descriptors. The existing studies applied only the MLR algorithm to develop models for predicting *K*_ca_ values and did not fully exploit any powerful nonlinear algorithms. In contrast, the QSPR models developed in this study showed excellent prediction accuracy while fitting more measured *K*_ca_ values. Further statistical analyses adequately demonstrated the reliability of these models. In addition, the nonlinear relationship between the plant cuticle–air partition coefficients and molecular descriptors of the compounds was established for the first time in this study.

## 3. Materials and Methods

### 3.1. Dataset Preparation

**Figure 5:**
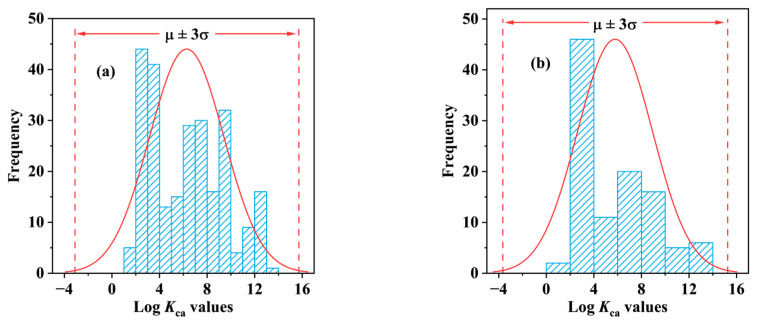
Distribution of experimental data of log *K*_ca_ values. (**a**) Distribution of 255 experimental log *K*_ca_ values for 106 compounds; (**b**) Distribution of average experimental log *K*_ca_ values for 106 compounds.

The data points for the experimental log *K*_ca_ dataset (dataset (I)), consisting of 255 data points measured for 106 compounds and 25 plant species, were collected from existing studies. Specifically, for compounds whose *K*_ca_ measured values had not been directly reported but their plant cuticle/water partition coefficient (*K*_cw_) and gas/water partition coefficients (*K*_aw_) had been reported, their log *K*_ca_ value was calculated as follows: log *K*_ca_ = log *K*_cw_ − log *K*_aw_ [10]. The plant species and tissue types used for the experimental measurements and the sources corresponding to each data point are listed in Table S7. The difference in the log *K*_ca_ values measured for each compound in different tissues of different plant species was very small, and the presence of these similar data points significantly increased the risk of data leakage. Therefore, the log *K*_ca_ values from different plant species and different tissue types were averaged in this study, resulting in dataset (II) containing the measured log *K*_ca_ values of 106 compounds (Table S8). The mean log *K*_ca_ values with standard deviation were 6.30 ± 3.14 for dataset (I) and 5.79 ± 3.15 for dataset (II). Following data quality testing (Figure 5), it was concluded that both dataset (I) and dataset (II) meet the Pauta criterion [57].

### 3.2. Descriptor Generation and Filtering

The molecular structure characterization information of the compounds was described through molecular descriptors [58]. After determining the molecular structures of the compounds, the geometric structures of the compounds were fully optimized using MM2 molecular mechanics [59]. A descriptor pool consisting of 5290 molecular descriptors was generated using alvaDesc software (Version 1.0.8) [60]. Preliminary descriptor screening was carried out in two steps, the first of which involved removing descriptors with missing, constant, and near-constant values, and the second of which involved identifying descriptors with pairwise correlations greater than 0.9 and removing one of the interrelated descriptors in order to reduce redundant information and make it easier to use the model in the future [61,62]. The remaining 165 and 163 descriptors of dataset (I) and dataset (II), respectively, were further filtered using the MLR algorithm in the next step. Detailed information about the steps of our further screening is presented below.

### 3.3. Model Development and Validation

The Y-ranking method was used to divide each dataset into a training set and a test set [63]. The training and test sets consisted of 80% and 20% log *K*_ca_ measured values and the corresponding molecular descriptors in the dataset, respectively [64,65]. The training sets were used to develop and internally validate each model, whereas the test set was solely dedicated to validating each model’s performance in predicting the *K*_ca_ for new compounds [66]. One linear algorithm (MLR) and three common nonlinear algorithms—MLP, KNN, and GBDT—were used to develop models for both datasets, resulting in 8 QSPR models. These 8 models were named in the format of “modeling algorithm + dataset number”. For example, the QSPR model developed using the KNN algorithm based on dataset (I) was named the KNN-1 model. Further details on these four machine-learning algorithms are provided in Text S1.

Identifying and selecting descriptors that contribute significantly to the dependent variable is essential for QSPR modeling. In this study, further screening of the descriptors was carried out using stepwise MLR in SPSS (Version 20.0) software [67]. Generally, the model developed using the best descriptor combination should have a high $R_{\mathrm{adj}}^{2}$ and $Q_{\mathrm{ext}}^{2}$ and the lowest possible number of descriptors [68]. Additionally, multicollinearity between descriptors was assessed using the variance inflation factor (*VIF*). The *VIF* of each descriptor should be less than 10 to avoid excessive inter-correlation between descriptors [69]. The MLR-1 and MLR-2 models were developed by establishing the relationship between measured log *K*_ca_ values and the best descriptor combinations based on dataset (I) and dataset (II), respectively.

The machine-learning library scikit-learn in Python (Version 3.9.6) was utilized to train the nonlinear QSPR models developed by the other three machine-learning algorithms [70]. With the help of the GridSearchCV function in the sklearn library, a grid search and five-fold cross-validation were performed to optimize various hyperparameters of models [71]. Table S1 lists these hyperparameters and their ranges for each model, as well as the modules for the different machine-learning algorithm implementations. Each modeling process of different ML algorithms took the best combination of descriptors screened by MLR as the independent variable to ensure consistency in the comparison among the models.

In accordance with the fourth principle of the OECD guidelines, assessing the fit, stability, and predictive performance of QSPR models with a wide range of internally and externally validated statistical parameters was essential for understanding the predictive quality of new compounds and ensuring the reliability of the developed models [72]. $R_{\mathrm{adj}}^{2}$, *MAE_tra_*, *RMSE*_tra_, and *s*_tra_ were used to measure the goodness of fit of the models [73,74]. Internal robustness was characterized by performing leave-one-out cross-validation and bootstrap cross-validation based on $Q_{\mathrm{LOO}}^{2}$ and $Q_{\mathrm{BOOT}}^{2}$ [75,76,77]. Each model’s external predictive ability was assessed based on $R_{\mathrm{ext}}^{2}$, $Q_{\mathrm{ext}}^{2}$, *CCC*, and three error-based metrics [78,79]. The leverage value method was employed to limit the compound structure space in which the model could reliably predict log *K*_ca_ [28]. In addition, Williams plots of leverage values (*h*_i_) versus normalized residuals (*δ*) were applied to visualize the applicability domains of the QSPR models. In addition, response outliers (the point with |*δ|* > 3) and structural outliers (the point with *h* > h*) could be clearly identified from the plots [80,81]. The formulas for *h*_i_, h*, and *δ* are presented in Text S3.

### 3.4. Model Interpretation

Understanding how dominant features affect model predictions is another important principle in QSPR model development [24]. The SHAP method was applied to explain the models developed using the GBDT algorithm and determine the effect of specific structural features of molecules on plant cuticles’ adsorption of airborne organic pollutants [82]. The SHAP value of a feature is determined by the average of the feature’s contribution across all possible feature alignments in the feature set [38]. It measures the degree and direction of the descriptor’s contribution to the prediction result: higher absolute SHAP values indicate a higher contribution, and whether a SHAP value demonstrates positivity or negativity corresponds to the positive and negative impact of the descriptor on the prediction result [83,84]. The global importance of a feature is reflected by averaging the absolute SHAP values corresponding to all the samples in that feature [85]. The formula for calculating SHAP values and more information about them are presented in Text S4.

## 4. Conclusions

*K*_ca_ is a key factor in assessing the capacity of plant cuticles to adsorb airborne organic pollutants. However, the development of reliable predictive tools to estimate *K*_ca_ has been hampered by the complexity of the molecular structures of organic pollutants. In this study, we established a comprehensive *K*_ca_ dataset that covers 255 experimental log *K*_ca_ values for 106 compounds in 25 plant species and 3 tissue types (dataset (I)). Additionally, 255 data points were averaged to form a second dataset (dataset (II)) containing 106 measured log *K*_ca_ values for 106 compounds. Based on these two datasets, eight QSPR models designed to predict *K*_ca_ values were developed using four machine-learning algorithms (MLR, MLP, KNN, and GBDT). Rigorous validation testing indicated that these models have acceptable fit, stability, and external predictive power. In addition, the GBDT-1 model ($R_{adj}^{2}$ = 0.995, $Q_{LOO}^{2}$ = 0.936, $Q_{BOOT}^{2}$ = 0.964, $R_{ext}^{2}$ = 0.911, $Q_{ext}^{2}$ = 0.902, and *CCC* = 0.952) and the GBDT-2 model ($R_{adj}^{2}$ = 0.925, $Q_{LOO}^{2}$ = 0.756, $Q_{BOOT}^{2}$ = 0.864, $R_{ext}^{2}$ = 0.837, $Q_{ext}^{2}$ = 0.811, and *CCC* = 0.891) showed the best performance on datasets (I) and (II), respectively. The GBDT-2 model can be recommended as the best tool for predicting *K*_ca_ values due to its low risk of data leakage. Interpreting the GBDT-1 and GBDT-2 models using the SHAP method revealed that molecular weight, molecular complexity, the number of hydrogen bond donors, the number of hydrogen bond acceptors, and polarizability are the most important factors that affect *K*_ca_ predictions. In summary, the models presented in this work provided a fast and reliable method for obtaining *K*_ca_ values, overcoming the obstacles of experimental challenges, halting kinetic models, and mistake-prone theoretical calculations. We hope that our findings will inspire the refinement of the modeling process to help with predicting other physicochemical properties using comparable workflows.

## Figures and Tables

**Table 1:** Descriptions involved in the final MLR models and corresponding statistical significance (*p*) and variance inflation factors (*VIF*) values.

Datasets	Descriptors	Parameters	*p*	*VIF*
(I)	*VE1_L*	coefficient sum of the last eigenvector (absolute values) from Laplace matrix	<0.001	7.527
	*LLS_02*	modified lead-like score from Monge et al. (8 rules)	<0.001	2.816
	*H_Dz(p)*	Harary-like index from Barysz matrix weighted by polarizability	<0.001	9.343
	*SpMax2_Bh(v)*	largest eigenvalue n. 2 of Burden matrix weighted by van der Waals volume	<0.001	2.937
(II)	*SpPos_A*	spectral positive sum from adjacency matrix	<0.001	6.594
	*LLS_02*	modified lead-like score from Monge et al. (8 rules)	<0.001	2.011
	*LLS_01*	modified lead-like score from Congreve et al. (6 rules)	<0.001	2.125
	*SpMax2_Bh(v)*	largest eigenvalue n. 2 of Burden matrix weighted by van der Waals volume	<0.001	3.128

Notes: Datasets (I): 255 experimental log *K*_ca_ values for 106 compounds; Datasets (II): average experimental log *K*_ca_ values for 106 compounds.

**Table 2:** Statistical parameters in the training and test sets for models of *K*_ca_.

Models	Training Set							Test Set						
	*n*_tra_	$R_{\mathrm{adj}}^{2}$	$Q_{\mathrm{LOO}}^{2}$	$Q_{\mathrm{BOOT}}^{2}$	*MAE*_tra_	*RMSE*_tra_	*s*_tra_	*n*_ext_	$R_{\mathrm{ext}}^{2}$	$Q_{\mathrm{ext}}^{2}$	*MAE*_ext_	*RMSE*_ext_	*s*_ext_	*CCC*
Threshold	-	>0.7	>0.6	>0.6	-	-	-	-	>0.7	>0.6	-	-	-	>0.85
MLR-1	204	0.873	0.869	0.872	0.874	1.101	1.114	51	0.839	0.835	1.024	1.296	1.364	0.914
MLP-1		0.850	0.678	0.676	0.917	1.194	1.209		0.790	0.784	1.009	1.485	1.564	0.889
KNN-1		0.920	0.742	0.778	0.638	0.871	0.882		0.859	0.855	0.706	1.214	1.279	0.925
GBDT-1		0.995	0.936	0.964	0.114	0.224	0.227		0.911	0.902	0.411	1.001	1.054	0.952
MLR-2	84	0.891	0.874	0.886	0.725	0.987	1.018	22	0.833	0.807	1.006	1.466	1.668	0.902
MLP-2		0.921	0.798	0.809	0.629	0.843	0.869		0.887	0.884	0.798	1.139	1.296	0.940
KNN-2		0.919	0.661	0.802	0.536	0.855	0.881		0.821	0.815	1.176	1.435	1.632	0.891
GBDT-2		0.925	0.756	0.864	0.504	0.821	0.847		0.837	0.811	1.097	1.451	1.651	0.891

Notes: *n*_tra_ and *n*_ext_: the number of chemicals in the training set and test set, respectively; $R_{\mathrm{adj}}^{2}$ and $R_{\mathrm{ext}}^{2}$: the correlation coefficient square between observed and fitted values in training set and test set, respectively; $Q_{\mathrm{LOO}}^{2}$: leave one out cross-validated *Q*^2^; $Q_{\mathrm{BOOT}}^{2}$: bootstrap method, 1/5, 5000 iterations; $Q_{\mathrm{ext}}^{2}$: external explained variance; *s_tra_*: standard error of estimate for training set; *s*_ext_: standard error of estimate for test set; *CCC*: concordance correlation coefficient.

## Data Availability

Data are contained within the article or Supplementary Material.

## Supplementary Materials

The following supporting information can be downloaded at: https://www.mdpi.com/article/10.3390/molecules29061381/s1, Text S1: The brief descriptions of the ML algorithms; Text S2: Statistical parameters; Text S3: Applicability domain; Text S4: Shapley value; Table S1: The hyperparameter optimization of each algorithm; Table S2: Observed log *K*_ca_ values in dataset (I). Predicted log *K*_ca_ values of the compounds by MLR-1, MLP-1, KNN-1 and GBDT-1 models. Values of descriptors used in QSPR models; Table S3: Observed log *K*_ca_ values in dataset (II). Predicted log *K*_ca_ values of the compounds by MLR-2, MLP-2, KNN-2 and GBDT-2 models. Values of descriptors used in QSPR models; Table S4: List of outliers for dataset (I); Table S5: List of outliers for dataset (II); Table S6: Comparison of the current models with previous models; Table S7: Values of log *K*_ca_ for compounds in dataset (I); Table S8: Values of log *K*_ca_ for compounds in dataset (II); Figure S1: The bar and line plots show the $R_{\mathrm{adj}}^{2}$ and $Q_{\mathrm{ext}}^{2}$ of the QSPR models. (a) Dataset (I); (b) Dataset (II); Figure S2: Application domain characterized by Williams plots: the MLP-1 (a), KNN-1 (b), GBDT-1 (c), MLR-2 (d), MLP-2 (e) and KNN-2 (f) models for log *K*_ca_; Figure S3: Cumulative distributions of residuals between the observed and predicted log *K*_ca_. (a) Dataset (I); (b) Dataset (II).
