# Supplementary material for: Machine-Learning-Based Prediction of Plant Cuticle–Air Partition Coefficients for Organic Pollutants: Revealing Mechanisms from a Molecular Structure Perspective
Source: Molecules. 2024 Mar 20;29(6):1381. doi: 10.3390/molecules29061381 (PMC10975432; doi:10.3390/molecules29061381)
Supplement: Supplementary file 1 [file molecules-29-01381-s001.zip › molecules-2901118-supplementary.pdf]

Supplementary information for

Machine-Learning-Based Prediction of Plant Cuticle–Air Partition Coefficients for Organic Pollutants: Revealing Mechanisms from a Molecular Structure Perspective

Tianyun Tao [1], Cuicui Tao [2], Tengyi Zhu [2,*]

[1] College of Agriculture, Yangzhou University, Yangzhou 225009, China.

[2] School of Environmental Science and Engineering, Yangzhou University, Yangzhou 225127, China.

* Correspondence: tyzhu@yzu.edu.cn

Number of pages for the supplementary information: 51

Number of texts: 4

Number of tables: 8

Number of figures: 3

* Reference style in supporting files is different than Journal format.

Contents

Texts..S2

Text S1. The brief descriptions of the ML algorithms...S2

Text S2. Statistical parameters...S3

Text S3. Applicability domain...S3

Text S4. Shapely value. S4

Tables...S6

Table S1. The hyperparameter optimization of each algorithm. S6

Table S2. Observed log K_{ca} values in dataset (I). Predicted log K_{ca} values of the compounds by MLR-1, MLP-1, KNN-1 and GBDT-1 models. Values of descriptors used in QSPR models...S7

Table S3. Observed log K_{ca} values in dataset (II). Predicted log K_{ca} values of the compounds by MLR-2, MLP-2, KNN-2 and GBDT-2 models. Values of descriptors used in QSPR models...S21

Table S4. List of outliers for dataset (I)...S26

Table S5. List of outliers for dataset (II). S27

Table S6. Comparison of the current models with previous models.........................S28

Table S7. Values of log K_{ca} for Compounds in dataset (I)...................................S29

Table S8. Values of log K_{ca} for Compounds in dataset (II). S42

Figures...S45

Figure. S1. The bar and line plots show the R^2_{adj} and Q^2_{ext} of the QSPR models. (a) Dataset (I); (b) Dataset (II). S45

Figure. S2. Application domain characterized by Williams plots: the MLP-1 (a), KNN-1 (b), GBDT-1 (c), MLR-2 (d), MLP-2 (e) and KNN-2 (f) models for log K_{ca}.........S46

Figure. S3. Cumulative distributions of residuals between the observed and predicted log K_{ca}. (a) Dataset (I); (b) Dataset (II)...S47

Reference S48

Texts

Text S1. The brief descriptions of the ML algorithms.

Multiple linear regression (MLR). MLR is the most commonly used machine learning method in QSAR modeling. The advantages of MLR are its simplicity and strong interpretability. The disadvantages of MLR are also obvious: (1) the number of descriptors that can maximally be used within an MLR is limited by the number of compounds; (2) MLR is prone to overfitting when there are too many redundant molecular descriptors.

Multi-Layer Perceptron (MLP). The most common ANN model is the Multi-Layer Perceptron (MLP). The MLP model is a flexible and general-purpose type of ANN composed of one input layer, one or more hidden layers, and one output layer. The MLP is a network formed by simple neurons called perceptron. The perceptron calculates a single output from multiple real-valued inputs by forming combinations of linear relationships according to input weights and even nonlinear transfer functions.

***k*-Nearest Neighbours (KNN).** KNN is one of the simplest methods of machine learning, the predicted activity of specific compound is determined by its *k* most similar compounds. Due to the simplicity of the algorithm, it often performs worse than some complex algorithms when faced with relatively complex problems (such as toxicity). The advantage of KNN lies in its ability to deal with nonlinear problems and its intuitive interpretability.

Gradient boosting decision tree (GBDT). The GBDT is a class of ensemble learning algorithms that combines several sequentially connected decision trees. Decision trees by themselves are occasionally referred to as relatively weak learners. However, in the case of GBDTs, by adding several decision trees in a series (e.g., boosting), whereby each subsequent tree minimizes the errors from the previous one,

the process of “boosting” becomes highly efficient, thus leading to the high accuracy of GBDT models.

Text S2. Statistical parameters.

Several statistical indices, including the Coefficient of Determination (R^2), the Root Mean Square Error (*RMSE*), the Mean Absolute Error (*MAE*) and the Mean Absolute Percentage Error (*MAPE*) of test set are employed to evaluate the performance of the proposed model, which are defined as:

$$R_{\text{ext}}^2 = 1 - \frac{\sum_{i=1}^{n_{\text{ext}}} (y_i - \bar{y}_i)^2 / (n\text{-}k\text{-}1)}{\sum_{i=1}^{n_{\text{ext}}} (y_i - \bar{y})^2 / (n\text{-}1)} \quad \text{(S1)}$$

$$RMSE_{\text{ext}} = \sqrt{\frac{\sum_{i}^{n_{\text{ext}}} (y_{\text{i}} - \bar{y}_{\text{i}})^2}{n_{ext}}} \quad \text{(S2)}$$

$$MAE_{ext} = \frac{\sum_{i}^{n_{ext}} | y_i - \bar{y}_i |}{n_{ext}} \quad \text{(S3)}$$

Where, n_{ext} is the number of data points in the test set; y_{i} and $\bar{y}_i$ are the measured and predicted values of PAH concentrations at the i-th data point in the test set, respectively; k is the number of input features; $\bar{y}$ is the average of the measured values of PAH concentrations in the test set.

Text S3. Applicability domain.

The applicability domain (AD) of the developed models was characterized by the Williams plot. In the Williams plots of standardized residuals (δ) versus leverage values (h), chemicals with the absolute values of standardized residual $|\delta| > 3$ were identified as outliers. The standardized residual (δ) was calculated by:

$$\delta = \frac{y_i - \hat{y}_i}{\sqrt{\sum_{i=1}^{n} (y_i - \hat{y}_i)^2 / (n - A - 1)}} \quad \text{(S4)}$$

where y_i and $\hat{y}_{\text{i}}$ are the observed value and predicted value for the i-th compound,

respectively; n is the number of compounds and A is the number of descriptors.

The measure of how far a chemical is from the domain of applicability of a model is its leverage value (Hat), h_i, defined as:

$$h_i = x_i^T (X^T X)^{-1} x_i \quad \text{(S5)}$$

where x_i is the descriptor vector of the i-th compound; x_i^T is the transpose of x_i; X is the descriptor matrix and X^T is the transpose of X.

The limit of application domain was quantified as the warning leverage value (h*), expressed as:

$$h^* = 3(k+1)/n \quad \text{(S6)}$$

where k is the number of predictor variables used in the model. If a compound in the training set has $h_i > h^*$, it means that the compound is very influential on the model. If a compound in the validation set has $h_i > h^*$, then the compound is structurally distant from the compounds used in the training set.

Text S4. Shapely value.

The SHAP algorithm proposed by [1] is an interpretation method based on Shapley values [2], which aims to explain individual predictions by calculating the contribution of each predictor. For each predicted sample, the model generates a predicted value, and the SHAP value is the value assigned to each feature in the sample [2]. Suppose an GBDT model where group N (with n features) is used to predict the output. In SHAP, the contribution of each feature (which is feature j) to the model output $f(N)$ is assigned according to its marginal contribution. Finally, based on several axioms to help fairly distribute the contribution of each feature, the SHAP value for feature X_j in a model is given by:

$$SHAP(X_j) = \sum_{s \in N} \frac{\mathrm{k}!(p-\mathrm{k}-1)!}{p!} [f(S \cup \{j\}) - f(S)] \quad \text{(S7)}$$

where p is the total number of features, N is a set of all possible combinations of features

excluding X_j, S is a feature set in N, $f(S)$ is the model prediction with features in S, and $f(S \cup \{j\})$ is the model prediction with features in S plus feature X_j. The interpretation of Eq. (S16) is that the SHAP value of a feature is its marginal contribution to model prediction averaged over all possible models with different combinations of features. SHAP value has a number of useful properties such as efficiency, symmetry, dummy and additivity [2]. The efficiency property refers to the requirement that all feature contributions total up to the difference between the prediction and the average. Symmetry means that two features have the same SHAP values if they contribute equally to the model. Dummy means that a feature has a SHAP value of zero if its marginal contribution to all possible models is zero. The property of additivity requires that the aggregate of predictions from individual models equal the prediction from the combination of all models. [2] proved that the solution is fair and unique if all four properties are satisfied.

In summary, the SHAP value is the Shapley value of the conditional expectation function of the original model. We applied SHAP values to account for the approximate contribution of predictors to predictions based on the average training output. Compared with the evaluation indicators of traditional models, SHAP value is more based on statistical theoretical knowledge and it is mainly affected by the performance of ML model. Due to ML models are very suitable for dealing with complex nonlinear relationship problems, it can provide new clues for some mechanistic problems.

Tables

Table S1. The hyperparameter optimization of each algorithm.

Algorithms	Parameters to be optimized	Optimal hyperparameters		Package
		Datasets (I)	Datasets (II)	
MLR	-	-	-	SPSS
MLP	activation: ('logistic', 'tanh', 'relu')<br>solver: ('sgd', 'adam')<br>hidden_layer_size: (Number of hidden layers: 1 ~ 5; Number of hidden layer nodes: 1 ~ 50)	activation = 'relu'<br>solver = 'adam'<br>hidden_layer_sizes = (Number of hidden layers = 1; Number of hidden layer nodes = 7)	activation = 'relu'<br>solver = 'sgd'<br>hidden_layer_sizes = (Number of hidden layers = 2; Number of hidden layer nodes = 13)	Sklearn package with neural_network module in Python
KNN	*n_neighbors*: (2 ~ 10, distance = 1)	*n_neighbors* = 9	*n_neighbors* = 2	Sklearn package with neighbors module in Python
GBDT	*n_estimators*: (50 ~ 500, distance = 50)<br>*learning_rate*: (0.05, 0.1, 0.15, 0.2)<br>*min_samples_leaf*: (5,10,15,20)<br>*max_depth*: (1 ~ 10, distance = 1)	*n_estimators* = 250<br>*learning_rate* = 0.2<br>*min_samples_leaf* = 10<br>*max_depth* = 8	*n_estimators* = 200<br>*learning_rate* = 0.1<br>*min_samples_leaf* = 10<br>*max_depth* = 3	Sklearn package with ensemble module in Python

Table S2. Observed log K_{ca} values in dataset (I). Predicted log K_{ca} values of the compounds by MLR-1, MLP-1, KNN-1 and GBDT-1 models. Values of descriptors used in QSPR models.

ID	CAS	Plant name	Tissue type	Descriptors				Observed	Predicted			
				VE1_L	LLS_02	H_Dz(p)	SpMax2_Bh(v)		MLR-1	MLP-1	KNN-1	GBDT-1
1	100-02-7	Capsicum (pepper)	CM	3.16	1.00	19.89	3.14	9.51	6.44	6.57	8.51	9.41
2	100-02-7	Capsicum (pepper)	MX	3.16	1.00	19.89	3.14	9.57	6.44	6.57	8.51	9.41
3	100-02-7	Citrus	CM	3.16	1.00	19.89	3.14	9.33	6.44	6.57	8.51	9.41
4	100-02-7	Citrus	MX	3.16	1.00	19.89	3.14	9.30	6.44	6.57	8.51	9.41
5	100-02-7	Ficus (rubber plant)	CM	3.16	1.00	19.89	3.14	9.34	6.44	6.57	8.51	9.41
6	100-02-7	Ficus (rubber plant)	MX	3.16	1.00	19.89	3.14	9.43	6.44	6.57	8.51	9.41
7	100-02-7	Lycopersicum esculentum Mill. (tomato fruit)	CM	3.16	1.00	19.89	3.14	9.43	6.44	6.57	8.51	9.41
8	100-02-7	Lycopersicum esculentum Mill. (tomato fruit)	MX	3.16	1.00	19.89	3.14	9.45	6.44	6.57	8.51	9.41
9	100-02-7	Prunus laurocerasus L. (cherry laurel leaf)	CM	3.16	1.00	19.89	3.14	9.31	6.44	6.57	8.51	9.41
10	100-17-4	Hordeum vulgare L.	Whole	3.32	1.00	21.81	3.18	6.09	6.89	6.99	8.67	6.04
11	100-17-4	Hordeum vulgare L.	Whole	3.32	1.00	21.81	3.18	5.92	6.89	6.99	8.67	6.04
12	100-41-4	Lycopersicum esculentum Mill. (tomato fruit)	MX	2.83	0.88	21.49	3.22	3.40	3.98	3.25	3.40	3.15
13	100-41-4	Lycopersicum esculentum Mill. (tomato fruit)	MX	2.83	0.88	21.49	3.22	3.31	3.98	3.25	3.40	3.15
14	100-42-5	Lycopersicum esculentum Mill. (tomato fruit)	MX	2.83	0.88	22.97	3.22	3.74	4.11	3.25	3.40	3.69
15	100-42-5	Lycopersicum esculentum Mill. (tomato fruit)	MX	2.83	0.88	22.97	3.22	3.63	4.11	3.25	3.40	3.69
16	101-42-8	Capsicum (pepper)	CM	3.46	1.00	26.64	3.38	9.01	7.43	7.61	8.74	9.11
17	101-42-8	Lycopersicum esculentum Mill. (tomato fruit)	CM	3.46	1.00	26.64	3.38	9.11	7.43	7.61	8.74	9.11

ID	CAS	Plant name	Tissue type	Descriptors				Observed	Predicted			
				VE1_L	LLS_02	H_Dz(p)	SpMax2_Bh(v)		MLR-1	MLP-1	KNN-1	GBDT-1
18	106-48-9	Capsicum (pepper)	CM	2.83	1.00	20.03	2.82	7.04	6.12	5.57	6.90	6.74
19	106-48-9	Malus domestica (apple)	CM	2.83	1.00	20.03	2.82	6.94	6.12	5.57-	6.90	6.74
20	106-48-9	Solanum lycopersicum (tomato fruit)	CM	2.83	1.00	20.03	2.82	6.92	6.12	5.57	6.90	6.74
21	106-48-9	Solanum tuberosum	CM	2.83	1.00	20.03	2.82	6.35	6.12	5.57	6.90	6.74
22	106-89-8	Lycopersicum esculentum Mill. (tomato fruit)	MX	2.24	1.00	5.11	2.68	3.34	3.68	3.68	3.16	3.34
23	106-89-8	Lycopersicum esculentum Mill. (tomato fruit)	MX	2.24	1.00	5.11	2.68	3.34	3.68	3.68	3.16	3.34
24	106-93-4	Lycopersicum esculentum Mill. (tomato fruit)	MX	2.00	0.88	7.04	2.33	3.46	1.89	2.10	2.80	3.37
25	106-93-4	Lycopersicum esculentum Mill. (tomato fruit)	MX	2.00	0.88	7.04	2.33	3.26	1.89	2.10	2.80	3.37
26	107-05-1	Lycopersicum esculentum Mill. (tomato fruit)	MX	2.00	0.88	6.08	2.55	2.08	1.56	1.98	2.73	2.40
27	107-05-1	Lycopersicum esculentum Mill. (tomato fruit)	MX	2.00	0.88	6.08	2.55	2.62	1.56	1.98	2.73	2.40
28	107-06-2	Lycopersicum esculentum Mill. (tomato fruit)	MX	2.00	0.88	5.35	2.25	2.77	1.81	2.16	2.78	2.77
29	107-06-2	Lycopersicum esculentum Mill. (tomato fruit)	MX	2.00	0.88	5.35	2.25	2.73	1.81	2.16	2.78	2.77
30	107-13-1	Lycopersicum esculentum Mill. (tomato fruit)	MX	2.00	1.00	6.09	2.55	2.40	3.44	3.07	2.81	2.15
31	107-13-1	Lycopersicum esculentum Mill. (tomato fruit)	MX	2.00	1.00	6.09	2.55	1.91	3.44	3.07	2.81	2.15
32	108-03-2	Lycopersicum esculentum Mill. (tomato fruit)	MX	2.45	1.00	3.63	3.00	3.40	3.63	4.36	3.30	3.38
33	108-03-2	Lycopersicum esculentum Mill. (tomato fruit)	MX	2.45	1.00	3.63	3.00	3.55	3.63	4.36	3.30	3.38
34	108-10-1	Lycopersicum esculentum Mill. (tomato fruit)	MX	2.65	1.00	10.47	3.19	3.04	4.47	5.15	3.61	2.97

ID	CAS	Plant name	Tissue type	Descriptors				Observed	Predicted			
				VE1_L	LLS_02	H_Dz(p)	SpMax2_Bh(v)		MLR-1	MLP-1	KNN-1	GBDT-1
35	108-10-1	Lycopersicum esculentum Mill. (tomato fruit)	MX	2.65	1.00	10.47	3.19	2.87	4.47	5.15	3.61	2.97
36	108-88-3	Lycopersicum esculentum Mill. (tomato fruit)	MX	2.65	0.88	18.39	3.00	3.15	3.54	3.01	3.50	3.06
37	108-88-3	Lycopersicum esculentum Mill. (tomato fruit)	MX	2.65	0.88	18.39	3.00	3.05	3.54	3.01	3.50	3.06
38	108-90-7	Lycopersicum esculentum Mill. (tomato fruit)	MX	2.65	0.88	19.08	2.78	3.52	3.84	3.12	3.17	3.57
39	108-94-1	Lycopersicum esculentum Mill. (tomato fruit)	MX	2.65	1.00	11.55	2.94	3.92	4.83	5.00	5.61	3.87
40	108-94-1	Lycopersicum esculentum Mill. (tomato fruit)	MX	2.65	1.00	11.55	2.94	3.75	4.83	5.00	5.61	3.87
41	108-95-2	Capsicum (pepper)	CM	2.65	1.00	15.76	2.76	6.44	5.42	4.99	5.94	6.58
42	108-95-2	Capsicum (pepper)	MX	2.65	1.00	15.76	2.76	6.52	5.42	4.99	5.94	6.58
43	108-95-2	Capsicum (pepper)	MX	2.65	1.00	15.76	2.76	7.18	5.42	4.99	5.94	6.58
44	108-95-2	Ficus (rubber plant)	CM	2.65	1.00	15.76	2.76	6.36	5.42	4.99	5.94	6.58
45	108-95-2	Ficus (rubber plant)	MX	2.65	1.00	15.76	2.76	6.54	5.42	4.99	5.94	6.58
46	108-95-2	Lycopersicum esculentum Mill. (tomato fruit)	CM	2.65	1.00	15.76	2.76	6.43	5.42	4.99	5.94	6.58
47	108-95-2	Lycopersicum esculentum Mill. (tomato fruit)	MX	2.65	1.00	15.76	2.76	6.49	5.42	4.99	5.94	6.58
48	109-60-4	Lycopersicum esculentum Mill. (tomato fruit)	MX	2.65	1.00	6.07	3.22	2.85	4.03	5.04	3.56	2.80
49	109-60-4	Lycopersicum esculentum Mill. (tomato fruit)	MX	2.65	1.00	6.07	3.22	2.76	4.03	5.04	3.56	2.80
50	109-99-9	Lycopersicum esculentum Mill. (tomato fruit)	MX	2.24	1.00	4.56	2.63	2.67	3.68	3.63	3.16	3.03
51	109-99-9	Lycopersicum esculentum Mill. (tomato fruit)	MX	2.24	1.00	4.56	2.63	3.32	3.68	3.63	3.16	3.03
52	110-82-7	Lycopersicum esculentum Mill. (tomato fruit)	MX	2.45	0.88	10.00	2.91	2.23	2.46	2.73	2.56	2.17

ID	CAS	Plant name	Tissue type	Descriptors				Observed	Predicted			
				VE1_L	LLS_02	H_Dz(p)	SpMax2_Bh(v)		MLR-1	MLP-1	KNN-1	GBDT-1
53	110-82-7	Lycopersicum esculentum Mill. (tomato fruit)	MX	2.45	0.88	10.00	2.91	2.34	2.46	2.73	2.56	2.17
54	110-86-1	Lycopersicum esculentum Mill. (tomato fruit)	MX	2.45	1.00	12.56	2.72	3.83	4.77	4.41	5.61	3.84
55	110-86-1	Lycopersicum esculentum Mill. (tomato fruit)	MX	2.45	1.00	12.56	2.72	3.78	4.77	4.41	5.61	3.84
56	111-27-3	Lycopersicum esculentum Mill. (tomato fruit)	MX	2.65	1.00	9.00	3.24	4.52	4.29	5.14	3.56	4.45
57	111-27-3	Lycopersicum esculentum Mill. (tomato fruit)	MX	2.65	1.00	9.00	3.24	4.45	4.29	5.14	3.56	4.45
58	117-81-7	Capsicum (pepper)	CM	5.29	0.75	85.96	3.68	12.18	12.61	12.17	11.21	12.16
59	117-81-7	Capsicum (pepper)	MX	5.29	0.75	85.96	3.68	12.36	12.61	12.17	11.21	12.16
60	117-81-7	Citrus	CM	5.29	0.75	85.96	3.68	11.92	12.61	12.17	11.21	12.16
61	117-81-7	Citrus	MX	5.29	0.75	85.96	3.68	12.08	12.61	12.17	11.21	12.16
62	117-81-7	Ficus (rubber plant)	CM	5.29	0.75	85.96	3.68	11.98	12.61	12.17	11.21	12.16
63	117-81-7	Ficus (rubber plant)	MX	5.29	0.75	85.96	3.68	12.28	12.61	12.17	11.21	12.16
64	117-81-7	Lycopersicum esculentum Mill. (tomato fruit)	CM	5.29	0.75	85.96	3.68	12.02	12.61	12.17	11.21	12.16
65	117-81-7	Lycopersicum esculentum Mill. (tomato fruit)	MX	5.29	0.75	85.96	3.68	12.03	12.61	12.17	11.21	12.16
66	118-74-1	Capsicum (pepper)	CM	3.46	0.75	44.97	2.89	7.07	5.92	5.71	6.37	7.00
67	118-74-1	Capsicum (pepper)	MX	3.46	0.75	44.97	2.89	6.78	5.92	5.71	6.37	7.00
68	118-74-1	Citrus	CM	3.46	0.75	44.97	2.89	6.97	5.92	5.71	6.37	7.00
69	118-74-1	Citrus	MX	3.46	0.75	44.97	2.89	7.06	5.92	5.71	6.37	7.00
70	118-74-1	Ficus (rubber plant)	CM	3.46	0.75	44.97	2.89	7.01	5.92	5.71	6.37	7.00
71	118-74-1	Ficus (rubber plant)	MX	3.46	0.75	44.97	2.89	7.28	5.92	5.71	6.37	7.00
72	118-74-1	Lycopersicum esculentum Mill. (tomato fruit)	CM	3.46	0.75	44.97	2.89	7.10	5.92	5.71	6.37	7.00

ID	CAS	Plant name	Tissue type	Descriptors				Observed	Predicted			
				VE1_L	LLS_02	H_Dz(p)	SpMax2_Bh(v)		MLR-1	MLP-1	KNN-1	GBDT-1
73	118-74-1	Lycopersicum esculentum Mill. (tomato fruit)	MX	3.46	0.75	44.97	2.89	6.85	5.92	5.71	6.37	7.00
74	118-74-1	Myriophyllum spicatum	Whole	3.46	0.75	44.97	2.89	4.30	5.92	5.71	6.37	7.00
75	118-96-7	Hordeum vulgare L.	Whole	4.00	1.00	35.49	3.29	8.59	9.43	8.94	9.69	8.57
76	118-96-7	Hordeum vulgare L.	Whole	4.00	1.00	35.49	3.29	8.59	9.43	8.94	9.69	8.57
77	119-27-7	Hordeum vulgare L.	Whole	3.74	1.00	27.79	3.26	7.88	8.22	8.13	8.42	7.85
78	119-27-7	Hordeum vulgare L.	Whole	3.74	1.00	27.79	3.26	7.81	8.22	8.13	8.42	7.85
79	120-12-7	Euonymus japonicus	CM	3.74	0.75	61.18	3.70	8.23	7.16	6.77	7.28	8.19
80	120-83-2	Capsicum (pepper)	CM	3.00	1.00	24.73	2.84	7.85	6.88	6.12	6.95	7.52
81	120-83-2	Malus domestica (apple)	CM	3.00	1.00	24.73	2.84	7.70	6.88	6.12	6.95	7.52
82	120-83-2	Solanum lycopersicum (tomato fruit)	CM	3.00	1.00	24.73	2.84	7.74	6.88	6.12	6.95	7.52
83	120-83-2	Solanum tuberosum	CM	3.00	1.00	24.73	2.84	7.14	6.88	6.12	6.95	7.52
84	121-14-2	Hordeum vulgare L.	Whole	3.61	1.00	28.38	3.28	7.09	7.98	7.88	8.43	7.05
85	121-14-2	Hordeum vulgare L.	Whole	3.61	1.00	28.38	3.28	7.00	7.98	7.88	8.43	7.05
86	123-86-4	Lycopersicum esculentum Mill. (tomato fruit)	MX	2.83	1.00	8.47	3.27	3.28	4.57	5.56	3.56	3.25
87	123-86-4	Lycopersicum esculentum Mill. (tomato fruit)	MX	2.83	1.00	8.47	3.27	3.16	4.57	5.56	3.56	3.25
88	123-91-1	Lycopersicum esculentum Mill. (tomato fruit)	MX	2.45	1.00	3.68	2.91	3.15	3.73	4.29	3.11	3.15
89	123-91-1	Lycopersicum esculentum Mill. (tomato fruit)	MX	2.45	1.00	3.68	2.91	3.14	3.73	4.29	3.11	3.15
90	126-73-8	Madagascar jasmine	CM	4.12	0.88	34.78	3.48	7.36	7.54	7.39	8.16	7.35
91	127-18-4	Lycopersicum esculentum Mill. (tomato fruit)	MX	2.45	0.88	13.92	2.06	2.98	3.71	3.16	2.69	2.97
92	129-00-0	Euonymus japonicus	CM	4.00	0.75	78.91	3.67	9.48	9.37	8.64	8.94	8.81
93	129-00-0	Pinus	CM	4.00	0.75	78.91	3.67	8.11	9.37	8.64	8.94	8.81

ID	CAS	Plant name	Tissue type	Descriptors				Observed	Predicted			
				VE1_L	LLS_02	H_Dz(p)	SpMax2_Bh(v)		MLR-1	MLP-1	KNN-1	GBDT-1
94	13029-08-8	Rhododendron L.	CM	3.74	0.75	55.46	3.68	5.00	6.64	6.52	7.10	5.09
95	138-86-3	Lycopersicum esculentum Mill. (tomato fruit)	MX	3.16	0.88	26.12	3.43	4.04	4.86	4.31	4.26	3.96
96	138-86-3	Lycopersicum esculentum Mill. (tomato fruit)	MX	3.16	0.88	26.12	3.43	3.86	4.86	4.31	4.26	3.96
97	141-78-6	Lycopersicum esculentum Mill. (tomato fruit)	MX	2.45	1.00	3.91	3.10	2.53	3.55	4.44	3.32	2.43
98	141-78-6	Lycopersicum esculentum Mill. (tomato fruit)	MX	2.45	1.00	3.91	3.10	2.29	3.55	4.44	3.32	2.43
99	142-82-5	Lycopersicum esculentum Mill. (tomato fruit)	MX	2.65	0.88	11.15	3.32	2.51	2.53	2.92	3.20	2.52
100	142-82-5	Lycopersicum esculentum Mill. (tomato fruit)	MX	2.65	0.88	11.15	3.32	2.56	2.53	2.92	3.20	2.52
101	150-68-5	Capsicum (pepper)	CM	3.61	1.00	31.60	3.38	9.30	8.18	8.07	9.23	9.25
102	150-68-5	Lycopersicum esculentum Mill. (tomato fruit)	CM	3.61	1.00	31.60	3.38	9.21	8.18	8.07	9.23	9.25
103	15545-48-9	Capsicum (pepper)	CM	3.74	1.00	36.36	3.39	9.80	8.89	8.52	9.92	9.83
104	15545-48-9	Lycopersicum esculentum Mill. (tomato fruit)	CM	3.74	1.00	36.36	3.39	9.73	8.89	8.52	9.92	9.83
105	1912-24-9	Capsicum (pepper)	CM	3.74	1.00	28.44	3.41	9.29	8.12	8.27	9.23	9.22
106	1912-24-9	Capsicum (pepper)	MX	3.74	1.00	28.44	3.41	9.30	8.12	8.27	9.23	9.22
107	1912-24-9	Citrus	CM	3.74	1.00	28.44	3.41	9.25	8.12	8.27	9.23	9.22
108	1912-24-9	Citrus	MX	3.74	1.00	28.44	3.41	9.27	8.12	8.27	9.23	9.22
109	1912-24-9	Ficus (rubber plant)	CM	3.74	1.00	28.44	3.41	9.26	8.12	8.27	9.23	9.22
110	1912-24-9	Ficus (rubber plant)	MX	3.74	1.00	28.44	3.41	9.25	8.12	8.27	9.23	9.22
111	1912-24-9	Lycopersicum esculentum Mill. (tomato fruit)	CM	3.74	1.00	28.44	3.41	9.22	8.12	8.27	9.23	9.22
112	1912-24-9	Lycopersicum esculentum Mill. (tomato fruit)	MX	3.74	1.00	28.44	3.41	9.23	8.12	8.27	9.23	9.22

ID	CAS	Plant name	Tissue type	Descriptors				Observed	Predicted			
				VE1_L	LLS_02	H_Dz(p)	SpMax2_Bh(v)		MLR-1	MLP-1	KNN-1	GBDT-1
113	1912-24-9	Prunus laurocerasus L. (cherry laurel leaf)	CM	3.74	1.00	28.44	3.41	9.00	8.12	8.27	9.23	9.22
114	191-24-2	Euonymus japonicus	CM	4.69	0.63	132.06	3.85	13.99	13.68	13.81	12.21	12.50
115	198-55-0	Capsicum (pepper)	CM	4.47	0.63	111.74	3.85	12.29	11.34	11.69	12.21	12.30
116	198-55-0	Capsicum (pepper)	MX	4.47	0.63	111.74	3.85	12.32	11.34	11.69	12.21	12.30
117	198-55-0	Citrus	CM	4.47	0.63	111.74	3.85	12.19	11.34	11.69	12.21	12.30
118	198-55-0	Citrus	MX	4.47	0.63	111.74	3.85	12.33	11.34	11.69	12.21	12.30
119	198-55-0	Ficus (rubber plant)	CM	4.47	0.63	111.74	3.85	11.94	11.34	11.69	12.21	12.30
120	198-55-0	Ficus (rubber plant)	MX	4.47	0.63	111.74	3.85	12.32	11.34	11.69	12.21	12.30
121	198-55-0	Lycopersicum esculentum Mill. (tomato fruit)	CM	4.47	0.63	111.74	3.85	12.24	11.34	11.69	12.21	12.30
122	198-55-0	Lycopersicum esculentum Mill. (tomato fruit)	MX	4.47	0.63	111.74	3.85	12.23	11.34	11.69	12.21	12.30
123	2050-23-9	Madagascar jasmine	CM	4.00	0.88	27.74	3.46	7.13	6.66	6.55	5.79	7.10
124	2051-24-3	Myriophyllum spicatum	Whole	4.69	0.50	112.31	3.75	6.15	10.06	13.01	12.21	11.83
125	2051-62-9	Rhododendron L.	CM	3.61	0.75	48.82	3.67	5.60	5.75	6.01	6.93	5.53
126	206-44-0	Euonymus japonicus	CM	4.00	0.75	74.31	3.69	9.33	8.92	8.27	7.72	9.21
127	21725-46-2	Capsicum (pepper)	CM	4.00	1.00	36.81	3.50	9.94	9.33	9.31	9.89	9.94
128	218-01-9	Euonymus japonicus	CM	4.24	0.75	91.08	3.85	11.20	10.81	10.30	8.94	11.15
129	26914-33-0	Myriophyllum spicatum	Whole	4.00	0.75	68.08	3.70	5.08	8.32	7.75	7.70	7.34
130	31508-00-6	Rhododendron L.	CM	4.12	0.75	73.74	3.71	7.80	9.09	8.46	8.03	7.83
131	330-54-1	Capsicum (pepper)	CM	3.74	1.00	37.11	3.39	10.45	8.96	8.54	9.92	10.43
132	330-54-1	Lycopersicum esculentum Mill. (tomato fruit)	CM	3.74	1.00	37.11	3.39	10.42	8.96	8.54	9.92	10.43
133	34123-59-6	Capsicum (pepper)	CM	3.87	1.00	39.08	3.43	10.82	9.36	9.06	9.89	10.79

ID	CAS	Plant name	Tissue type	Descriptors				Observed	Predicted			
				VE1_L	LLS_02	H_Dz(p)	SpMax2_Bh(v)		MLR-1	MLP-1	KNN-1	GBDT-1
134	34123-59-6	Lycopersicum esculentum Mill. (tomato fruit)	CM	3.87	1.00	39.08	3.43	10.76	9.36	9.06	9.89	10.79
135	34123-59-6	Myriophyllum spicatum	whole	3.87	1.00	39.08	3.43	9.94	9.36	9.06	9.89	10.79
136	35065-28-2	Rhododendron L.	CM	4.24	0.75	81.04	3.72	7.60	10.00	9.30	8.94	7.60
137	35065-29-3	Rhododendron L.	CM	4.36	0.75	88.24	3.73	9.19	10.90	10.12	8.94	9.18
138	35693-99-3	Rhododendron L.	CM	4.00	0.75	67.76	3.70	6.60	8.29	7.72	7.32	6.71
139	37680-73-2	Rhododendron L.	CM	4.12	0.75	74.25	3.71	7.20	9.14	8.50	8.03	7.91
140	42576-02-3	Capsicum (pepper)	CM	4.69	1.00	69.51	3.80	9.80	13.47	13.77	10.97	9.88
141	50-32-8	Euonymus japonicus	CM	4.47	0.63	110.52	3.87	12.55	11.20	11.61	12.21	12.30
142	53-70-3	Euonymus japonicus	CM	4.69	0.63	122.61	3.97	12.79	12.66	13.14	12.21	12.50
143	56-23-5	Lycopersicum esculentum Mill. (tomato fruit)	MX	2.24	0.88	9.44	1.09	2.43	3.88	3.27	2.81	2.48
144	56-23-5	Lycopersicum esculentum Mill. (tomato fruit)	MX	2.24	0.88	9.44	1.09	2.44	3.88	3.27	2.81	2.48
145	56-55-3	Euonymus japonicus	CM	4.24	0.75	90.55	3.86	11.16	10.75	10.27	8.94	11.15
146	6032-29-7	Lycopersicum esculentum Mill. (tomato fruit)	MX	2.45	1.00	6.73	3.10	3.68	3.82	4.52	3.33	3.67
147	608-93-5	Myriophyllum spicatum	whole	3.32	0.75	39.06	2.89	4.68	5.07	5.21	6.37	4.68
148	626-93-7	Lycopersicum esculentum Mill. (tomato fruit)	MX	2.65	1.00	9.17	3.24	4.08	4.30	5.14	3.56	4.08
149	626-93-7	Lycopersicum esculentum Mill. (tomato fruit)	MX	2.65	1.00	9.17	3.24	4.08	4.30	5.14	3.56	4.08
150	63-25-2	Prunus laurocerasus L. (cherry laurel leaf)	CM	3.87	1.00	45.76	3.39	9.63	10.03	9.48	9.89	9.65
151	64-10-8	Capsicum (pepper)	CM	3.16	1.00	21.94	3.18	9.73	6.59	6.66	9.01	9.62
152	64-17-5	Hedera helix L.	CM	1.73	1.00	0.57	2.16	2.74	2.78	2.72	2.71	2.75
153	64-17-5	Lycopersicum esculentum Mill. (tomato fruit)	MX	1.73	1.00	0.57	2.16	2.81	2.78	2.72	2.71	2.75

ID	CAS	Plant name	Tissue type	Descriptors				Observed	Predicted			
				VE1_L	LLS_02	H_Dz(p)	SpMax2_Bh(v)		MLR-1	MLP-1	KNN-1	GBDT-1
154	64-17-5	Lycopersicum esculentum Mill. (tomato fruit)	MX	1.73	1.00	0.57	2.16	2.81	2.78	2.72	2.71	2.75
155	65-85-0	Capsicum (pepper)	MX	3.00	1.00	21.15	3.07	6.68	6.31	6.18	6.78	6.76
156	65-85-0	Chenopodium album	MX	3.00	1.00	21.15	3.07	6.86	6.31	6.18	6.78	6.76
157	65-85-0	Ginkgo biloba L. (ginkgo leaf)	CM	3.00	1.00	21.15	3.07	6.82	6.31	6.18	6.78	6.76
158	65-85-0	Hedera helix L.	CM	3.00	1.00	21.15	3.07	6.64	6.31	6.18	6.78	6.76
159	65-85-0	Juglans regia L. (English walnut leaf)	CM	3.00	1.00	21.15	3.07	6.82	6.31	6.18	6.78	6.76
160	65-85-0	Prunus laurocerasus L. (cherry laurel leaf)	CM	3.00	1.00	21.15	3.07	6.78	6.31	6.18	6.78	6.76
161	67-56-1	Citrus	CM	1.41	1.00	-0.75	1.41	2.59	2.81	2.46	2.93	2.66
162	67-56-1	Lycopersicum esculentum Mill. (tomato fruit)	MX	1.41	1.00	-0.75	1.41	2.66	2.81	2.46	2.93	2.66
163	67-56-1	Lycopersicum esculentum Mill. (tomato fruit)	MX	1.41	1.00	-0.75	1.41	2.62	2.81	2.46	2.93	2.66
164	67-63-0	Lycopersicum esculentum Mill. (tomato fruit)	MX	2.00	1.00	2.38	2.57	2.88	3.07	3.06	2.81	2.69
165	67-64-1	Lycopersicum esculentum Mill. (tomato fruit)	MX	2.00	1.00	3.16	2.57	2.40	3.14	3.05	2.81	2.69
166	67-64-1	Lycopersicum esculentum Mill. (tomato fruit)	MX	2.00	1.00	3.16	2.57	1.99	3.14	3.05	2.81	2.69
167	67-66-3	Lycopersicum esculentum Mill. (tomato fruit)	MX	2.00	0.88	6.15	1.09	2.63	3.10	2.80	2.75	2.63
168	67-66-3	Lycopersicum esculentum Mill. (tomato fruit)	MX	2.00	0.88	6.15	1.09	2.76	3.10	2.80	2.75	2.63
169	69-72-7	Ginkgo biloba L. (ginkgo leaf)	CM	3.16	1.00	22.53	3.07	7.42	6.77	6.59	6.90	7.33
170	69-72-7	Hedera helix L.	CM	3.16	1.00	22.53	3.07	7.09	6.77	6.59	6.90	7.33
171	69-72-7	Juglans regia L. (English walnut leaf)	CM	3.16	1.00	22.53	3.07	7.37	6.77	6.59	6.90	7.33

ID	CAS	Plant name	Tissue type	Descriptors				Observed	Predicted			
				VE1_L	LLS_02	H_Dz(p)	SpMax2_Bh(v)		MLR-1	MLP-1	KNN-1	GBDT-1
172	69-72-7	Prunus laurocerasus L. (cherry laurel leaf)	CM	3.16	1.00	22.53	3.07	7.48	6.77	6.59	6.90	7.33
173	69-79-4	Hedera helix L.	CM	4.80	0.63	42.33	3.59	4.19	5.75	8.93	6.77	4.28
174	7012-37-5	Rhododendron L.	CM	3.87	0.75	60.71	3.69	6.60	7.38	6.97	7.28	6.59
175	71-23-8	Lycopersicum esculentum Mill. (tomato fruit)	MX	2.00	1.00	2.31	2.63	3.24	3.00	3.03	2.83	3.20
176	71-23-8	Lycopersicum esculentum Mill. (tomato fruit)	MX	2.00	1.00	2.31	2.63	3.26	3.00	3.03	2.83	3.20
177	71-36-3	Lycopersicum esculentum Mill. (tomato fruit)	MX	2.24	1.00	4.33	2.92	3.70	3.36	3.83	3.11	3.65
178	71-36-3	Lycopersicum esculentum Mill. (tomato fruit)	MX	2.24	1.00	4.33	2.92	3.66	3.36	3.83	3.11	3.65
179	71-41-0	Lycopersicum esculentum Mill. (tomato fruit)	MX	2.45	1.00	6.58	3.11	4.10	3.80	4.52	3.32	4.06
180	71-41-0	Lycopersicum esculentum Mill. (tomato fruit)	MX	2.45	1.00	6.58	3.11	4.04	3.80	4.52	3.32	4.06
181	71-43-2	Lolium multiflorum Lam. (annual rye grass)	Whole	2.45	0.88	15.00	2.72	2.73	3.13	2.78	2.67	2.65
182	71-43-2	Lycopersicum esculentum Mill. (tomato fruit)	MX	2.45	0.88	15.00	2.72	2.63	3.13	2.78	2.67	2.65
183	71-43-2	Lycopersicum esculentum Mill. (tomato fruit)	MX	2.45	0.88	15.00	2.72	2.60	3.13	2.78	2.67	2.65
184	71-55-6	Lycopersicum esculentum Mill. (tomato fruit)	MX	2.24	0.88	8.81	2.26	2.58	2.59	2.63	2.78	2.61
185	71-55-6	Lycopersicum esculentum Mill. (tomato fruit)	MX	2.24	0.88	8.81	2.26	2.62	2.59	2.63	2.78	2.61
186	75-05-8	Lycopersicum esculentum Mill. (tomato fruit)	MX	1.73	1.00	2.93	2.14	2.58	3.02	2.70	2.71	2.66
187	75-05-8	Lycopersicum esculentum Mill. (tomato fruit)	MX	1.73	1.00	2.93	2.14	2.65	3.02	2.70	2.71	2.66
188	75-09-2	Lycopersicum esculentum Mill. (tomato fruit)	MX	1.73	0.88	3.48	1.09	2.38	2.31	2.37	2.83	3.81

ID	CAS	Plant name	Tissue type	Descriptors				Observed	Predicted			
				VE1_L	LLS_02	H_Dz(p)	SpMax2_Bh(v)		MLR-1	MLP-1	KNN-1	GBDT-1
189	75-09-2	Lycopersicum esculentum Mill. (tomato fruit)	MX	1.73	0.88	3.48	1.09	5.38	2.31	2.37	2.83	3.81
190	75-35-4	Lycopersicum esculentum Mill. (tomato fruit)	MX	2.00	0.88	7.01	1.80	1.86	2.43	2.39	2.69	2.01
191	75-35-4	Lycopersicum esculentum Mill. (tomato fruit)	MX	2.00	0.88	7.01	1.80	2.01	2.43	2.39	2.69	2.01
192	75-65-0	Lycopersicum esculentum Mill. (tomato fruit)	MX	2.24	1.00	4.69	2.57	2.91	3.76	3.59	2.97	2.88
193	75-65-0	Lycopersicum esculentum Mill. (tomato fruit)	MX	2.24	1.00	4.69	2.57	2.93	3.76	3.59	2.97	2.88
194	75-85-4	Lycopersicum esculentum Mill. (tomato fruit)	MX	2.45	1.00	7.10	2.96	3.36	4.00	4.43	3.30	3.38
195	76-06-2	Lycopersicum esculentum Mill. (tomato fruit)	MX	2.65	1.00	8.48	2.48	3.28	5.03	4.57	4.35	3.19
196	76-06-2	Lycopersicum esculentum Mill. (tomato fruit)	MX	2.65	1.00	8.48	2.48	3.13	5.03	4.57	4.35	3.19
197	76738-62-0	Capsicum (pepper)	CM	4.47	1.00	64.65	3.71	11.75	12.67	12.76	10.97	11.85
198	76738-62-0	Lycopersicum esculentum Mill. (tomato fruit)	CM	4.47	1.00	64.65	3.71	11.98	12.67	12.76	10.97	11.85
199	76738-62-0	Pyrus communis (pears)	CM	4.47	1.00	64.65	3.71	11.89	12.67	12.76	10.97	11.85
200	76738-62-0	Vanilla sp.	CM	4.47	1.00	64.65	3.71	11.02	12.67	12.76	10.97	11.85
201	77-74-7	Lycopersicum esculentum Mill. (tomato fruit)	MX	2.65	1.00	9.75	3.09	3.69	4.51	5.05	3.57	3.69
202	78-79-5	Lycopersicum esculentum Mill. (tomato fruit)	MX	2.24	0.88	9.40	2.91	1.59	1.97	2.26	2.56	1.62
203	78-79-5	Lycopersicum esculentum Mill. (tomato fruit)	MX	2.24	0.88	9.40	2.91	1.59	1.97	2.26	2.56	1.62
204	78-83-1	Lycopersicum esculentum Mill. (tomato fruit)	MX	2.24	1.00	4.54	2.65	3.41	3.66	3.64	3.16	3.38
205	78-87-5	Lycopersicum esculentum Mill. (tomato fruit)	MX	2.24	0.88	7.76	2.65	2.89	2.09	2.43	2.35	3.04

ID	CAS	Plant name	Tissue type	Descriptors				Observed	Predicted			
				VE1_L	LLS_02	H_Dz(p)	SpMax2_Bh(v)		MLR-1	MLP-1	KNN-1	GBDT-1
206	78-87-5	Lycopersicum esculentum Mill. (tomato fruit)	MX	2.24	0.88	7.76	2.65	3.09	2.09	2.43	2.35	3.04
207	78-92-2	Lycopersicum esculentum Mill. (tomato fruit)	MX	2.24	1.00	4.45	2.90	3.29	3.39	3.82	3.22	3.28
208	78-92-2	Lycopersicum esculentum Mill. (tomato fruit)	MX	2.24	1.00	4.45	2.90	3.22	3.39	3.82	3.22	3.28
209	78-93-3	Lycopersicum esculentum Mill. (tomato fruit)	MX	2.24	1.00	5.32	2.90	2.56	3.47	3.84	3.22	2.43
210	78-93-3	Lycopersicum esculentum Mill. (tomato fruit)	MX	2.24	1.00	5.32	2.90	2.43	3.47	3.84	3.22	2.43
211	79-01-6	Lycopersicum esculentum Mill. (tomato fruit)	MX	2.24	0.88	10.15	1.97	2.88	3.03	2.78	2.69	2.90
212	79-01-6	Lycopersicum esculentum Mill. (tomato fruit)	MX	2.24	0.88	10.15	1.97	2.96	3.03	2.78	2.69	2.90
213	83-32-9	Euonymus japonicus	CM	3.46	0.88	46.65	3.37	6.58	7.45	6.45	5.75	7.40
214	85-01-8	Capsicum (pepper)	MX	3.74	0.75	61.71	3.66	7.66	7.25	6.80	7.28	7.24
215	85-01-8	Euonymus japonicus	CM	3.74	0.75	61.71	3.66	7.99	7.25	6.80	7.28	7.24
216	85-01-8	Lolium multiflorum Lam. (annual rye grass)	Whole	3.74	0.75	61.71	3.66	6.46	7.25	6.80	7.28	7.24
217	85-01-8	Malus domestica (apple)	MX	3.74	0.75	61.71	3.66	7.56	7.25	6.80	7.28	7.24
218	85-01-8	Pinus	CM	3.74	0.75	61.71	3.66	6.62	7.25	6.80	7.28	7.24
219	85-01-8	Solanum lycopersicum (tomato fruit)	MX	3.74	0.75	61.71	3.66	7.54	7.25	6.80	7.28	7.24
220	85-01-8	Solanum tuberosum	MX	3.74	0.75	61.71	3.66	7.10	7.25	6.80	7.28	7.24
221	85-01-8	Vitis heyneana Roem. Et Schult	MX	3.74	0.75	61.71	3.66	7.39	7.25	6.80	7.28	7.24
222	87-86-5	Capsicum (pepper)	CM	3.46	1.00	41.02	2.89	8.32	9.29	7.69	7.82	8.25
223	87-86-5	Capsicum (pepper)	MX	3.46	1.00	41.02	2.89	8.38	9.29	7.69	7.82	8.25
224	87-86-5	Citrus	CM	3.46	1.00	41.02	2.89	8.08	9.29	7.69	7.82	8.25

ID	CAS	Plant name	Tissue type	Descriptors				Observed	Predicted			
				VE1_L	LLS_02	H_Dz(p)	SpMax2_Bh(v)		MLR-1	MLP-1	KNN-1	GBDT-1
225	87-86-5	Citrus	MX	3.46	1.00	41.02	2.89	8.12	9.29	7.69	7.82	8.25
226	87-86-5	Ficus (rubber plant)	CM	3.46	1.00	41.02	2.89	8.21	9.29	7.69	7.82	8.25
227	87-86-5	Ficus (rubber plant)	MX	3.46	1.00	41.02	2.89	8.26	9.29	7.69	7.82	8.25
228	87-86-5	Lycopersicum esculentum Mill. (tomato fruit)	CM	3.46	1.00	41.02	2.89	8.23	9.29	7.69	7.82	8.25
229	87-86-5	Lycopersicum esculentum Mill. (tomato fruit)	MX	3.46	1.00	41.02	2.89	8.36	9.29	7.69	7.82	8.25
230	88-06-2	Capsicum (pepper)	CM	3.16	1.00	29.75	2.85	6.92	7.68	6.64	7.17	6.55
231	88-06-2	Malus domestica (apple)	CM	3.16	1.00	29.75	2.85	6.67	7.68	6.64	7.17	6.55
232	88-06-2	Solanum lycopersicum (tomato fruit)	CM	3.16	1.00	29.75	2.85	6.93	7.68	6.64	7.17	6.55
233	88-06-2	Solanum tuberosum	CM	3.16	1.00	29.75	2.85	6.17	7.68	6.64	7.17	6.55
234	88-75-5	Capsicum (pepper)	CM	3.16	1.00	20.00	3.13	5.36	6.46	6.57	7.60	5.39
235	88-75-5	Capsicum (pepper)	MX	3.16	1.00	20.00	3.13	5.48	6.46	6.57	7.60	5.39
236	88-75-5	Ficus (rubber plant)	CM	3.16	1.00	20.00	3.13	5.28	6.46	6.57	7.60	5.39
237	88-75-5	Ficus (rubber plant)	MX	3.16	1.00	20.00	3.13	5.43	6.46	6.57	7.60	5.39
238	88-75-5	Lycopersicum esculentum Mill. (tomato fruit)	CM	3.16	1.00	20.00	3.13	5.27	6.46	6.57	7.60	5.39
239	88-75-5	Lycopersicum esculentum Mill. (tomato fruit)	MX	3.16	1.00	20.00	3.13	5.43	6.46	6.57	7.60	5.39
240	90-15-3	Capsicum (pepper)	MX	3.32	1.00	37.57	3.37	8.80	8.17	7.61	8.75	8.86
241	90-15-3	Capsicum (pepper)	MX	3.32	1.00	37.57	3.37	8.88	8.17	7.61	8.75	8.86
242	90-15-3	Malus domestica (apple)	MX	3.32	1.00	37.57	3.37	8.91	8.17	7.61	8.75	8.86
243	90-15-3	Solanum lycopersicum (tomato fruit)	MX	3.32	1.00	37.57	3.37	8.78	8.17	7.61	8.75	8.86
244	91-20-3	Capsicum (pepper)	MX	3.16	0.88	35.85	3.37	5.10	5.83	4.90	4.38	5.12
245	91-20-3	Capsicum (pepper)	MX	3.16	0.88	35.85	3.37	5.12	5.83	4.90	4.38	5.12

ID	CAS	Plant name	Tissue type	Descriptors				Observed	Predicted			
				VE1_L	LLS_02	H_Dz(p)	SpMax2_Bh(v)		MLR-1	MLP-1	KNN-1	GBDT-1
246	91-20-3	Malus domestica (apple)	MX	3.16	0.88	35.85	3.37	5.15	5.83	4.90	4.38	5.12
247	91-20-3	Solanum lycopersicum (tomato fruit)	MX	3.16	0.88	35.85	3.37	5.11	5.83	4.90	4.38	5.12
248	95-47-6	Lycopersicum esculentum Mill. (tomato fruit)	MX	2.83	0.88	22.16	3.03	3.56	4.24	3.35	3.50	3.60
249	95-47-6	Lycopersicum esculentum Mill. (tomato fruit)	MX	2.83	0.88	22.16	3.03	3.64	4.24	3.35	3.50	3.60
250	95-50-1	Festuca rubra L. (red fescu)	Whole	2.83	0.88	23.60	2.81	4.05	4.60	3.46	3.32	4.04
251	95-50-1	Lolium arundinaceium (tall fescu)	Whole	2.83	0.88	23.60	2.81	4.06	4.60	3.46	3.32	4.04
252	95-50-1	Lolium multiflorum Lam. (annual rye grass)	Whole	2.83	0.88	23.60	2.81	4.06	4.60	3.46	3.32	4.04
253	95-50-1	Spinacia oleracea (spinach)	Whole	2.83	0.88	23.60	2.81	3.96	4.60	3.46	3.32	4.04
254	95-94-3	Myriophyllum spicatum	Whole	3.16	0.75	33.42	2.89	3.63	4.23	4.71	6.37	3.66
255	99607-70-2	Hedera helix L.	CM	4.80	1.00	70.84	3.63	12.21	13.98	13.90	10.97	12.17

Table S3. Observed log K_{ca} values in dataset (II). Predicted log K_{ca} values of the compounds by MLR-2, MLP-2, KNN-2 and GBDT-2 models. Values of descriptors used in QSPR models.

ID	CAS	Descriptors				Observed	Predicted			
		SpPos_A	LLS_02	LLS_01	SpMax2_Bh(v)		MLR-2	MLP-2	KNN-2	GBDT-2
1	100-02-7	5.97	1.00	0.67	3.14	9.41	4.65	5.57	6.61	5.67
2	100-17-4	6.81	1.00	0.83	3.18	6.01	6.95	7.06	7.46	7.98
3	100-41-4	5.21	0.88	1.00	3.22	3.36	4.47	4.17	3.52	3.53
4	100-42-5	5.21	0.88	1.00	3.22	3.68	4.46	4.17	3.52	3.64
5	101-42-8	7.28	1.00	1.00	3.38	9.06	8.50	8.53	9.05	9.18
6	106-48-9	4.96	1.00	1.00	2.82	6.81	6.28	5.93	7.21	5.98
7	106-89-8	3.21	1.00	1.00	2.68	3.34	4.17	3.75	3.38	3.44
8	106-93-4	2.24	0.88	1.00	2.33	3.36	1.87	2.37	2.67	2.54
9	107-05-1	2.24	0.88	1.00	2.55	2.35	1.53	2.15	2.67	2.54
10	107-06-2	2.24	0.88	1.00	2.25	2.75	1.98	2.44	2.67	2.54
11	107-13-1	2.24	1.00	1.00	2.55	2.15	3.08	2.87	2.54	2.80
12	108-03-2	3.08	1.00	1.00	3.00	3.48	3.50	3.11	3.42	3.14
13	108-10-1	3.41	1.00	1.00	3.19	2.95	3.66	3.38	2.88	3.27
14	108-88-3	4.36	0.88	1.00	3.00	3.10	3.67	3.35	3.35	3.22
15	108-90-7	4.36	0.88	1.00	2.78	3.52	4.00	3.65	3.09	4.09
16	108-94-1	4.36	1.00	1.00	2.94	3.84	5.29	4.92	3.49	4.62
17	108-95-2	4.36	1.00	1.00	2.76	6.57	5.57	5.17	6.69	5.14
18	109-60-4	3.86	1.00	1.00	3.22	2.80	4.22	3.89	3.44	3.27
19	109-99-9	3.24	1.00	1.00	2.63	3.00	4.27	3.83	3.38	3.44
20	110-82-7	4.00	0.88	0.83	2.91	2.29	2.09	2.57	3.57	2.32
21	110-86-1	4.00	1.00	1.00	2.72	3.80	5.16	4.75	4.95	3.57

ID	CAS	Descriptors				Observed	Predicted			
		**SpPos_A**	**LLS_02**	**LLS_01**	**SpMax2_Bh(v)**		**MLR-2**	**MLP-2**	**KNN-2**	**GBDT-2**
22	111-27-3	4.03	1.00	0.83	3.24	4.48	3.17	4.20	3.85	4.06
23	117-81-7	17.50	0.75	0.33	3.68	12.11	13.58	12.64	8.15	9.22
24	118-74-1	7.30	0.75	0.83	2.89	6.71	4.95	5.17	5.70	5.96
25	118-96-7	9.19	1.00	0.67	3.29	8.59	8.70	9.24	8.22	8.20
26	119-27-7	8.41	1.00	0.67	3.26	7.85	7.71	8.35	8.22	8.20
27	120-12-7	9.66	0.75	0.83	3.70	8.23	6.85	6.95	6.95	7.10
28	120-83-2	5.40	1.00	1.00	2.84	7.61	6.82	6.51	7.21	7.60
29	121-14-2	7.58	1.00	0.67	3.28	7.05	6.58	7.32	8.22	8.07
30	123-86-4	4.38	1.00	0.83	3.27	3.22	3.59	4.32	3.85	3.79
31	123-91-1	4.00	1.00	1.00	2.91	3.14	4.86	4.47	3.49	3.27
32	126-73-8	9.97	0.88	0.50	3.48	7.36	6.68	6.39	8.18	5.72
33	127-18-4	3.00	0.88	1.00	2.06	2.98	3.28	3.04	2.86	2.96
34	129-00-0	11.25	0.75	0.83	3.67	8.79	9.01	9.18	9.06	8.80
35	13029-08-8	9.08	0.75	0.83	3.68	5.00	6.12	6.26	6.45	6.72
36	138-86-3	5.97	0.88	0.83	3.43	3.95	3.92	3.81	4.53	3.94
37	141-78-6	3.08	1.00	1.00	3.10	2.41	3.35	3.04	3.04	3.09
38	142-82-5	4.03	0.88	0.67	3.32	2.53	0.28	2.77	4.53	2.29
39	150-68-5	7.84	1.00	1.00	3.38	9.26	9.24	9.27	9.84	9.40
40	15545-48-9	8.30	1.00	1.00	3.39	9.77	9.83	9.87	10.10	10.01
41	1912-24-9	8.08	1.00	0.50	3.41	9.23	5.81	6.88	8.54	8.92
42	191-24-2	15.71	0.63	0.83	3.85	13.99	13.10	13.43	13.11	11.47
43	198-55-0	14.12	0.63	0.83	3.85	12.23	11.00	11.52	12.39	11.47
44	2050-23-9	9.42	0.88	0.67	3.46	7.13	7.20	7.13	6.86	6.02
45	2051-24-3	13.66	0.50	0.67	3.75	6.15	7.76	7.83	9.19	9.56

ID	CAS	Descriptors				Observed	Predicted			
		SpPos_A	LLS_02	LLS_01	SpMax2_Bh(v)		MLR-2	MLP-2	KNN-2	GBDT-2
46	2051-62-9	8.57	0.75	0.83	3.67	5.60	5.45	5.63	6.45	5.98
47	206-44-0	11.25	0.75	0.83	3.69	9.33	8.98	9.15	9.06	9.39
48	21725-46-2	9.26	1.00	0.50	3.50	9.94	7.23	8.10	8.91	8.36
49	218-01-9	12.60	0.75	0.83	3.85	11.20	10.52	10.87	11.18	11.47
50	26914-33-0	10.33	0.75	0.83	3.70	5.08	7.74	7.82	5.84	7.16
51	31508-00-6	10.59	0.75	0.67	3.71	7.80	6.83	6.48	7.50	7.56
52	330-54-1	8.30	1.00	1.00	3.39	10.44	9.84	9.88	10.10	10.17
53	34123-59-6	8.89	1.00	1.00	3.43	10.51	10.55	10.61	10.14	10.45
54	35065-28-2	11.11	0.75	0.67	3.72	7.60	7.51	7.19	7.70	7.56
55	35065-29-3	11.75	0.75	0.67	3.73	9.19	8.34	8.06	8.39	9.56
56	35693-99-3	10.14	0.75	0.83	3.70	6.60	7.50	7.57	5.84	7.16
57	37680-73-2	10.58	0.75	0.67	3.71	7.20	6.83	6.48	7.50	6.53
58	42576-02-3	13.67	1.00	0.17	3.80	9.80	10.17	10.83	9.51	9.55
59	50-32-8	14.11	0.63	0.83	3.87	12.55	10.95	11.49	12.39	11.47
60	53-70-3	15.44	0.63	0.83	3.97	12.79	12.55	12.96	13.27	11.47
61	56-23-5	2.00	0.88	1.00	1.09	2.43	3.44	3.41	3.16	2.54
62	56-55-3	12.55	0.75	0.83	3.86	11.16	10.44	10.80	11.18	11.47
63	6032-29-7	3.08	1.00	1.00	3.10	3.68	3.35	3.04	3.04	3.22
64	608-93-5	6.57	0.75	0.83	2.89	4.68	3.98	4.23	5.70	5.03
65	626-93-7	3.86	1.00	1.00	3.24	4.08	4.19	3.89	3.44	3.40
66	63-25-2	9.77	1.00	1.00	3.39	9.63	11.78	11.83	10.07	9.75
67	64-10-8	6.03	1.00	1.00	3.18	9.73	7.14	7.06	8.54	8.79
68	64-17-5	1.41	1.00	1.00	2.16	2.79	2.57	2.62	2.70	2.80
69	65-85-0	5.59	1.00	1.00	3.07	6.77	6.74	6.57	7.05	7.12

ID	CAS	Descriptors				Observed	Predicted			
		SpPos_A	LLS_02	LLS_01	SpMax2_Bh(v)		MLR-2	MLP-2	KNN-2	GBDT-2
70	67-56-1	1.00	1.00	1.00	1.41	2.62	3.17	3.08	3.25	2.80
71	67-63-0	1.73	1.00	1.00	2.57	2.88	2.38	2.50	2.54	2.80
72	67-64-1	1.73	1.00	1.00	2.57	2.19	2.38	2.50	2.54	2.80
73	67-66-3	1.73	0.88	1.00	1.09	2.70	3.08	3.31	3.16	2.54
74	69-72-7	5.97	1.00	1.00	3.07	7.34	7.24	7.09	7.05	7.20
75	69-79-4	14.11	0.63	0.17	3.59	4.19	6.46	4.54	8.15	7.05
76	7012-37-5	9.46	0.75	0.83	3.69	6.60	6.60	6.71	6.95	7.42
77	71-23-8	2.24	1.00	1.00	2.63	3.25	2.96	2.81	3.17	2.98
78	71-36-3	2.73	1.00	1.00	2.92	3.68	3.17	2.84	3.08	3.14
79	71-41-0	3.49	1.00	1.00	3.11	4.07	3.89	3.52	3.05	3.22
80	71-43-2	4.00	0.88	1.00	2.72	2.65	3.62	3.29	3.09	3.15
81	71-55-6	2.00	0.88	1.00	2.26	2.60	1.66	2.29	2.67	2.54
82	75-05-8	1.41	1.00	1.00	2.14	2.62	2.60	2.64	2.70	2.80
83	75-09-2	1.41	0.88	1.00	1.09	3.88	2.66	3.18	3.16	2.54
84	75-35-4	1.73	0.88	1.00	1.80	1.94	2.00	2.56	2.46	2.54
85	75-65-0	2.00	1.00	1.00	2.57	2.92	2.73	2.69	2.56	2.80
86	75-85-4	2.91	1.00	1.00	2.96	3.36	3.34	2.96	3.42	3.14
87	76-06-2	3.30	1.00	1.00	2.48	3.21	4.60	4.10	2.68	3.25
88	76738-62-0	12.24	1.00	0.50	3.71	11.66	10.87	11.72	8.91	9.55
89	77-74-7	3.80	1.00	1.00	3.09	3.69	4.32	3.95	3.68	3.35
90	78-79-5	2.61	0.88	1.00	2.91	1.59	1.48	2.01	2.29	2.13
91	78-83-1	2.61	1.00	1.00	2.65	3.41	3.43	3.05	3.38	3.06
92	78-87-5	2.61	0.88	1.00	2.65	2.99	1.88	2.29	2.67	2.80
93	78-92-2	2.61	1.00	1.00	2.90	3.26	3.05	2.79	2.87	2.98

ID	CAS	Descriptors				Observed	Predicted			
		SpPos_A	LLS_02	LLS_01	SpMax2_Bh(v)		MLR-2	MLP-2	KNN-2	GBDT-2
94	78-93-3	2.61	1.00	1.00	2.90	2.49	3.05	2.79	2.87	2.76
95	79-01-6	2.61	0.88	1.00	1.97	2.92	2.92	2.92	2.46	2.61
96	83-32-9	8.31	0.88	0.83	3.37	6.58	7.11	7.26	5.85	6.51
97	85-01-8	9.72	0.75	0.83	3.66	7.29	7.00	7.09	6.95	6.77
98	87-86-5	7.30	1.00	0.83	2.89	8.25	8.03	8.11	7.46	8.94
99	88-06-2	5.82	1.00	0.83	2.85	6.67	6.14	6.19	7.46	6.67
100	88-75-5	5.97	1.00	0.67	3.13	5.38	4.68	5.58	6.61	5.67
101	90-15-3	7.25	1.00	1.00	3.37	8.84	8.47	8.50	9.05	9.18
102	91-20-3	6.84	0.88	0.83	3.37	5.12	5.16	5.16	4.53	4.95
103	95-47-6	4.98	0.88	1.00	3.03	3.60	4.44	4.10	3.35	3.71
104	95-50-1	4.98	0.88	0.83	2.81	4.03	3.54	3.37	3.78	4.18
105	95-94-3	5.84	0.75	0.83	2.89	3.63	3.02	3.41	5.70	4.29
106	99607-70-2	14.44	1.00	0.33	3.63	12.21	12.68	13.28	9.51	9.24

Table S4. List of outliers for dataset (I).

Models	ID	Chemicals	CAS number	Plant name	Tissue type
MLR-1	3	4-Nitrophenol	100-02-7	Citrus	CM
	8	4-Nitrophenol	100-02-7	Lycopersicum esculentum Mill. (tomato fruit)	MX
	124	Decachlorobiphenyl	2051-24-3	Myriophyllum spicatum	Whole
	129	Tetrachlorobiphenyl	26914-33-0	Myriophyllum spicatum	Whole
	140	bifenox	42576-02-3	*Capsicum* (pepper)	CM
MLP-1	3	4-Nitrophenol	100-02-7	Citrus	CM
	8	4-Nitrophenol	100-02-7	Lycopersicum esculentum Mill. (tomato fruit)	MX
	124	Decachlorobiphenyl	2051-24-3	Myriophyllum spicatum	Whole
	129	Tetrachlorobiphenyl	26914-33-0	Myriophyllum spicatum	Whole
	140	bifenox	42576-02-3	*Capsicum* (pepper)	CM
	173	maltose	69-79-4	Hedera helix L.	CM
KNN-1	11	4-Nitroanisole	100-17-4	Hordeum vulgare L.	Whole
	40	Cyclohexanone	108-94-1	Lycopersicum esculentum Mill. (tomato fruit)	MX
	74	Hexachlorobenzene	118-74-1	Myriophyllum spicatum	Whole
	124	Decachlorobiphenyl	2051-24-3	Myriophyllum spicatum	Whole
	129	Tetrachlorobiphenyl	26914-33-0	Myriophyllum spicatum	Whole
	238	2-Nitrophenol	88-75-5	Lycopersicum esculentum Mill. (tomato fruit)	CM
	239	2-Nitrophenol	88-75-5	Lycopersicum esculentum Mill. (tomato fruit)	MX
	254	1,2,4,5-Tetrachlorobenzene	95-94-3	Myriophyllum spicatum	Whole
GBDT-1	74	Hexachlorobenzene	118-74-1	Myriophyllum spicatum	Whole
	93	Pyrene	129-00-0	Pinus	CM
	124	Decachlorobiphenyl	2051-24-3	Myriophyllum spicatum	Whole
	129	Tetrachlorobiphenyl	26914-33-0	Myriophyllum spicatum	Whole
	188	Dichloromethane	75-09-2	Lycopersicum esculentum Mill. (tomato fruit)	MX
	189	Dichloromethane	75-09-2	Lycopersicum esculentum Mill. (tomato fruit)	MX
	215	Phenanthrene	85-01-8	Euonymus japonicus	CM
	216	Phenanthrene	85-01-8	Lolium multiflorum Lam. (annual rye grass)	Whole

Notes: The ID number refers to the ID number in Table S1.

Table S5. List of outliers for dataset (II).

Models	ID	Chemicals	CAS number
MLR-2	1	4-Nitrophenol	100-02-7
	41	Atrazine	1912-24-9
	48	Cyanazine	21725-46-2
MLP-2	1	4-Nitrophenol	100-02-7
	48	Cyanazine	21725-46-2
	56	PCB 52	35693-99-3
	67	Phenylurea	64-10-8
KNN-2	1	4-Nitrophenol	100-02-7
	23	bis(2-Ethylhexyl) phthalate	117-81-7
	45	Decachlorobiphenyl	2051-24-3
	75	maltose	69-79-4
	88	Paclobutrazol	76738-62-0
	106	cloquintocet-mexyl	99607-70-2
GBDT-2	1	4-Nitrophenol	100-02-7
	23	bis(2-Ethylhexyl) phthalate	117-81-7
	45	Decachlorobiphenyl	2051-24-3
	75	maltose	69-79-4
	106	cloquintocet-mexyl	99607-70-2

Notes: The ID number refers to the ID number in Table S2.

Table S6. Comparison of the current models with previous models.

Descriptors	*k*	*n*	Algorithms	R^2	*RMSE*	se	Reference
bp	1	50	MLR	0.829	-	0.260	[7]
$p°$	1	50	MLR	0.899	-	0.199	[7]
K_{oa}	1	38	MLR	0.819	-	0.237	[7]
HBD, P_L, P_H, $^3\chi_c$, MR	5	49	MLR	0.812	-	0.271	[7]
π_2^H, $\Sigma\alpha_2^H$, $\Sigma\beta_2^H$, L^{16}	4	62	MLR	0.994	-	0.230	[9]
E, *S*, *A*, *B*, *V*	5	215	MLR	0.995	-	0.223	[3]
E, *S*, *A*, *B*, *V*	5	105	MLR	0.996	-	0.172	[3]
VE1_L, *LLS_02*, *H_Dz(p)*, *SpMax2_Bh(v)*	4	255	MLR, MLP, KNN, GBDT	0.850~0.995	0.224~1.194	-	This study (dataset (I))
SpPos_A, *LLS_02*, *LLS_01*, *SpMax2_Bh(v)*	4	106	MLR, MLP, KNN, GBDT	0.891~0.925	0.821~0.987	-	This study (dataset (II))

Notes: *k* is the number of parameters; *n* is the number of total data set, se is the standard error, *bp* is the boiling points, $p°$ is the saturation vapor pressures, K_{oa} is 1-octanol/air (K_{oa}) partition coefficients, *HBD* is the hydrogen-bond donor descriptor, P_L is substructure lipophilic, P_H is substructure hydrophilic, $^3\chi_c$ is the third-order cluster index, *MR* is molar refraction, π_2^H is the VOC dipolarity/polariz ability, $\Sigma\alpha_2^H$ is the VOC hydrogen bond acidity, $\Sigma\beta_2^H$ is the VOC hydrogen bond basicity, L^{16} is the gas−hexadecane partition coefficient, *E* is the solute excess molar refractivity in units of (cm^3 mol^{-1})/10, *S* is the solute's dipolarity/polarity descriptor, *A* is characterization of molecular H-bond donor ability, *B* is describes the H-bond acceptor properties, *V* is McGowan_Volume.

Table S7. Values of log K_{ca} for Compounds in dataset (I).

ID	**Compounds**	**CAS**	**Plant name**	**Tissue type**	**Log K_{ca}**	**Ref**	**Log K_{aw}**	**Ref**	**Log K_{cw}**	**Ref**
1	4-Nitrophenol	100-02-7	*Capsicum* (pepper)	CM	9.51	[3,4]	-7.54	[3,4]	1.97	[4]
2	4-Nitrophenol	100-02-7	*Capsicum* (pepper)	MX	9.57	[3,4]	-7.54	[3,4]	2.03	[4]
3	4-Nitrophenol	100-02-7	Citrus	CM	9.33	[3,4]	-7.54	[3,4]	1.79	[4]
4	4-Nitrophenol	100-02-7	Citrus	MX	9.30	[3,4]	-7.54	[3,4]	1.76	[4]
5	4-Nitrophenol	100-02-7	Ficus (rubber plant)	CM	9.34	[3,4]	-7.54	[3,4]	1.80	[4]
6	4-Nitrophenol	100-02-7	Ficus (rubber plant)	MX	9.43	[3,4]	-7.54	[3,4]	1.89	[4]
7	4-Nitrophenol	100-02-7	Lycopersicum esculentum Mill. (tomato fruit)	CM	9.43	[3,4]	-7.54	[3,4]	1.89	[4]
8	4-Nitrophenol	100-02-7	Lycopersicum esculentum Mill. (tomato fruit)	MX	9.45	[3,4]	-7.54	[3,4]	1.91	[4]
9	4-Nitrophenol	100-02-7	Prunus laurocerasus L. (cherry laurel leaf)	CM	9.31	[3,5]	-7.54	[3,4]	1.77	[5]
10	4-Nitroanisole	100-17-4	Hordeum vulgare L.	Whole	6.09	[3,6]	-4.08	[3,6]	2.01	[6]
11	4-Nitroanisole	100-17-4	Hordeum vulgare L.	Whole	5.92	[3,6]	-4.08	[3,6]	1.84	[6]
12	Ethylbenzene	100-41-4	Lycopersicum esculentum Mill. (tomato fruit)	MX	3.40	[7]	-	-	-	-
13	Ethylbenzene	100-41-4	Lycopersicum esculentum Mill. (tomato fruit)	MX	3.31	[8,9]	-0.49	[8]	2.82	[9]
14	Styrene	100-42-5	Lycopersicum esculentum Mill. (tomato fruit)	MX	3.74	[7]	-0.80	[7]	2.95	[7]
15	Styrene	100-42-5	Lycopersicum esculentum Mill. (tomato fruit)	MX	3.63	[7,9]	-0.80	[7]	2.83	[9]
16	Fenuron	101-42-8	*Capsicum* (pepper)	CM	9.01	[3,10]	-8.41	[3,10]	0.60	[10]
17	Fenuron	101-42-8	Lycopersicum esculentum Mill. (tomato fruit)	CM	9.11	[3,10]	-8.41	[3,10]	0.70	[10]
18	4-Chlorophenol	106-48-9	*Capsicum* (pepper)	CM	7.04	[8,11]	-4.85	[8]	2.19	[11]
19	4-Chlorophenol	106-48-9	Malus domestica (apple)	CM	6.94	[8,11]	-4.85	[8]	2.09	[11]
20	4-Chlorophenol	106-48-9	Solanum lycopersicum (tomato fruit)	CM	6.92	[8,11]	-4.85	[8]	2.07	[11]

ID	Compounds	CAS	Plant name	Tissue type	Log K_{ca}	Ref	Log K_{aw}	Ref	Log K_{cw}	Ref
21	4-Chlorophenol	106-48-9	Solanum tuberosum	CM	6.35	[8,11]	-4.85	[8]	1.50	[11]
22	Epichlorohydrin	106-89-8	Lycopersicum esculentum Mill. (tomato fruit)	MX	3.34	[7]	-2.85	[7]	0.48	[7]
23	Epichlorohydrin	106-89-8	Lycopersicum esculentum Mill. (tomato fruit)	MX	3.34	[7,9]	-2.85	[7]	0.49	[9]
24	1,2-Dibromoethane	106-93-4	Lycopersicum esculentum Mill. (tomato fruit)	MX	3.46	[7]	-1.51	[7]	1.96	[7]
25	1,2-Dibromoethane	106-93-4	Lycopersicum esculentum Mill. (tomato fruit)	MX	3.26	[7,9]	-1.51	[7]	1.75	[9]
26	allyl chloride	107-05-1	Lycopersicum esculentum Mill. (tomato fruit)	MX	2.08	[7]	-0.96	[7]	1.12	[7]
27	allyl chloride	107-05-1	Lycopersicum esculentum Mill. (tomato fruit)	MX	2.62	[7,9]	-0.96	[7]	1.66	[9]
28	1,2-Dichloroethane	107-06-2	Lycopersicum esculentum Mill. (tomato fruit)	MX	2.77	[7]	-1.27	[7]	1.51	[7]
29	1,2-Dichloroethane	107-06-2	Lycopersicum esculentum Mill. (tomato fruit)	MX	2.73	[7,9]	-1.27	[7]	1.46	[9]
30	Acrylonitrile	107-13-1	Lycopersicum esculentum Mill. (tomato fruit)	MX	2.40	[7]	-1.89	[7]	0.51	[7]
31	Acrylonitrile	107-13-1	Lycopersicum esculentum Mill. (tomato fruit)	MX	1.91	[7,9]	-1.89	[7]	0.02	[9]
32	1-Nitropropane	108-03-2	Lycopersicum esculentum Mill. (tomato fruit)	MX	3.40	[7]	-2.60	[7]	0.79	[7]
33	1-Nitropropane	108-03-2	Lycopersicum esculentum Mill. (tomato fruit)	MX	3.55	[7,9]	-2.60	[7]	0.95	[9]
34	4-Methyl-2-pentanone	108-10-1	Lycopersicum esculentum Mill. (tomato fruit)	MX	3.04	[7]	-2.07	[7]	0.97	[7]
35	4-Methyl-2-pentanone	108-10-1	Lycopersicum esculentum Mill. (tomato fruit)	MX	2.87	[7,9]	-2.07	[7]	0.80	[9]
36	Toluene	108-88-3	Lycopersicum esculentum Mill. (tomato fruit)	MX	3.15	[7]	-0.55	[7]	2.60	[7]
37	Toluene	108-88-3	Lycopersicum esculentum Mill. (tomato fruit)	MX	3.05	[7,9]	-0.55	[7]	2.50	[9]
38	Chlorobenzene	108-90-7	Lycopersicum esculentum Mill. (tomato fruit)	MX	3.52	[7]	-0.82	[7]	2.70	[7]

ID	Compounds	CAS	Plant name	Tissue type	Log K_{ca}	Ref	Log K_{aw}	Ref	Log K_{cw}	Ref
39	Cyclohexanone	108-94-1	Lycopersicum esculentum Mill. (tomato fruit)	MX	3.92	[7]	-	-	-	-
40	Cyclohexanone	108-94-1	Lycopersicum esculentum Mill. (tomato fruit)	MX	3.75	[8,9]	-3.43	[8]	0.32	[9]
41	Phenol	108-95-2	*Capsicum* (pepper)	CM	6.44	[3,4]	-4.85	[3,4]	1.59	[4]
42	Phenol	108-95-2	*Capsicum* (pepper)	MX	6.52	[3,4]	-4.85	[3,4]	1.67	[4]
43	phenol	108-95-2	*Capsicum* (pepper)	MX	7.18	[3,4,12]	-4.85	[3,4]	2.33	[12]
44	Phenol	108-95-2	Ficus (rubber plant)	CM	6.36	[3,4]	-4.85	[3,4]	1.51	[4]
45	Phenol	108-95-2	Ficus (rubber plant)	MX	6.54	[3,4]	-4.85	[3,4]	1.69	[4]
46	Phenol	108-95-2	Lycopersicum esculentum Mill. (tomato fruit)	CM	6.43	[3,4]	-4.85	[3,4]	1.58	[4]
47	Phenol	108-95-2	Lycopersicum esculentum Mill. (tomato fruit)	MX	6.49	[3,4]	-4.85	[3,4]	1.64	[4]
48	propyl acetate	109-60-4	Lycopersicum esculentum Mill. (tomato fruit)	MX	2.85	[7]	-1.96	[7]	0.88	[7]
49	propyl acetate	109-60-4	Lycopersicum esculentum Mill. (tomato fruit)	MX	2.76	[7,9]	-1.96	[7]	0.80	[9]
50	Tetrahydrofuran	109-99-9	Lycopersicum esculentum Mill. (tomato fruit)	MX	2.67	[7]	-3.20	[7]	-0.52	[7]
51	Tetrahydrofuran	109-99-9	Lycopersicum esculentum Mill. (tomato fruit)	MX	3.32	[7,9]	-3.20	[7]	0.12	[9]
52	Cyclohexane	110-82-7	Lycopersicum esculentum Mill. (tomato fruit)	MX	2.23	[7]	-	-	-	-
53	Cyclohexane	110-82-7	Lycopersicum esculentum Mill. (tomato fruit)	MX	2.34	[8,9]	0.79	[8]	3.13	[9]
54	Pyridine	110-86-1	Lycopersicum esculentum Mill. (tomato fruit)	MX	3.83	[7]	-3.39	[7]	0.43	[7]
55	Pyridine	110-86-1	Lycopersicum esculentum Mill. (tomato fruit)	MX	3.78	[7,9]	-3.39	[7]	0.39	[9]
56	1-Hexanol	111-27-3	Lycopersicum esculentum Mill. (tomato fruit)	MX	4.52	[7,13]	-3.16	[7]	1.36	[13]
57	1-Hexanol	111-27-3	Lycopersicum esculentum Mill. (tomato fruit)	MX	4.45	[7,9]	-3.16	[7]	1.29	[9]

ID	**Compounds**	**CAS**	**Plant name**	**Tissue type**	**Log K_{ca}**	**Ref**	**Log K_{aw}**	**Ref**	**Log K_{cw}**	**Ref**
58	bis(2-Ethylhexyl) phthalate	117-81-7	*Capsicum* (pepper)	CM	12.18	[3,4]	-4.70	[3,4]	7.48	[4]
59	bis(2-Ethylhexyl) phthalate	117-81-7	*Capsicum* (pepper)	MX	12.36	[3,4]	-4.70	[3,4]	7.66	[4]
60	bis(2-Ethylhexyl) phthalate	117-81-7	Citrus	CM	11.92	[3,4]	-4.70	[3,4]	7.22	[4]
61	bis(2-Ethylhexyl) phthalate	117-81-7	Citrus	MX	12.08	[3,4]	-4.70	[3,4]	7.38	[4]
62	bis(2-Ethylhexyl) phthalate	117-81-7	Ficus (rubber plant)	CM	11.98	[3,4]	-4.70	[3,4]	7.28	[4]
63	bis(2-Ethylhexyl) phthalate	117-81-7	Ficus (rubber plant)	MX	12.28	[3,4]	-4.70	[3,4]	7.58	[4]
64	bis(2-Ethylhexyl) phthalate	117-81-7	Lycopersicum esculentum Mill. (tomato fruit)	CM	12.02	[3,4]	-4.70	[3,4]	7.32	[4]
65	bis(2-Ethylhexyl) phthalate	117-81-7	Lycopersicum esculentum Mill. (tomato fruit)	MX	12.03	[3,4]	-4.70	[3,4]	7.33	[4]
66	Hexachlorobenzene	118-74-1	*Capsicum* (pepper)	CM	7.07	[4,14]	-1.27	[14]	5.80	[4]
67	Hexachlorobenzene	118-74-1	*Capsicum* (pepper)	MX	6.78	[4,14]	-1.27	[14]	5.82	[4]
68	Hexachlorobenzene	118-74-1	Citrus	CM	6.97	[4,14]	-1.27	[14]	5.70	[4]
69	Hexachlorobenzene	118-74-1	Citrus	MX	7.06	[4,14]	-1.27	[14]	5.79	[4]
70	Hexachlorobenzene	118-74-1	Ficus (rubber plant)	CM	7.01	[4,14]	-1.27	[14]	5.74	[4]
71	Hexachlorobenzene	118-74-1	Ficus (rubber plant)	MX	7.28	[4,14]	-1.27	[14]	6.01	[4]
72	Hexachlorobenzene	118-74-1	Lycopersicum esculentum Mill. (tomato fruit)	CM	7.10	[4,14]	-1.27	[14]	5.83	[4]
73	Hexachlorobenzene	118-74-1	Lycopersicum esculentum Mill. (tomato fruit)	MX	6.85	[4,14]	-1.27	[14]	5.58	[4]
74	Hexachlorobenzene	118-74-1	Myriophyllum spicatum	Whole	4.30	[14,15]	-1.27	[14]	3.03	[15]
75	TNT	118-96-7	Hordeum vulgare L.	Whole	8.59	[3,6]	-6.54	[3,6]	2.05	[6]
76	TNT	118-96-7	Hordeum vulgare L.	Whole	8.59	[3,6]	-6.54	[3,6]	2.05	[6]
77	2,4-Dinitroanisole	119-27-7	Hordeum vulgare L.	Whole	7.88	[3,6]	-5.96	[3,6]	1.92	[6]
78	2,4-Dinitroanisole	119-27-7	Hordeum vulgare L.	Whole	7.81	[3,6]	-5.96	[3,6]	1.85	[6]

ID	**Compounds**	**CAS**	**Plant name**	**Tissue type**	**Log K_{ca}**	**Ref**	**Log K_{aw}**	**Ref**	**Log K_{cw}**	**Ref**
79	Anthracene	120-12-7	Euonymus japonicus	CM	8.23	[3,16]	-3.03	[3,16]	5.20	[16]
80	2,4-Dichlorophenol	120-83-2	*Capsicum* (pepper)	CM	7.85	[3,6,11]	-5.09	[3,6]	2.76	[11]
81	2,4-Dichlorophenol	120-83-2	Malus domestica (apple)	CM	7.70	[3,6,11]	-5.09	[3,6]	2.60	[11]
82	2,4-Dichlorophenol	120-83-2	Solanum lycopersicum (tomato fruit)	CM	7.74	[3,6,11]	-5.09	[3,6]	2.65	[11]
83	2,4-Dichlorophenol	120-83-2	Solanum tuberosum	CM	7.14	[3,6,11]	-5.09	[3,6]	2.05	[11]
84	2,4-Dinitrotoluene	121-14-2	Hordeum vulgare L.	Whole	7.09	[3,6]	-5.09	[3,6]	2.00	[6]
85	2,4-Dinitrotoluene	121-14-2	Hordeum vulgare L.	Whole	7.00	[3,6]	-5.09	[3,6]	1.91	[6]
86	Butyl acetate	123-86-4	Lycopersicum esculentum Mill. (tomato fruit)	MX	3.28	[7]	-1.82	[7]	1.45	[7]
87	Butyl acetate	123-86-4	Lycopersicum esculentum Mill. (tomato fruit)	MX	3.16	[7,9]	-1.82	[7]	1.34	[9]
88	1,4-Dioxane	123-91-1	Lycopersicum esculentum Mill. (tomato fruit)	MX	3.15	[7]	-3.70	[7]	-0.55	[7]
89	1,4-Dioxane	123-91-1	Lycopersicum esculentum Mill. (tomato fruit)	MX	3.14	[7,9]	-3.70	[7]	-0.56	[9]
90	Tributyl phosphate	126-73-8	Madagascar jasmine	CM	7.36	[3,17]	-4.82	[3,17]	2.54	[17]
91	Tetrachloroethylene	127-18-4	Lycopersicum esculentum Mill. (tomato fruit)	MX	2.98	[7]	-0.15	[7]	2.83	[7]
92	Pyrene	129-00-0	Euonymus japonicus	CM	9.48	[3,16]	-3.50	[3,16]	5.98	[16]
93	Pyrene	129-00-0	Pinus	CM	8.11	[3,16,18]	-3.50	[3,16]	4.61	[18]
94	PCB 4	13029-08-8	Rhododendron L.	CM	5.00	[19]	-	-	3.60	[19]
95	Limonene	138-86-3	Lycopersicum esculentum Mill. (tomato fruit)	MX	4.04	[7]	0.23	[7]	4.26	[7]
96	Limonene	138-86-3	Lycopersicum esculentum Mill. (tomato fruit)	MX	3.86	[7,9]	0.23	[7]	4.09	[9]
97	Ethyl acetate	141-78-6	Lycopersicum esculentum Mill. (tomato fruit)	MX	2.53	[7]	-1.92	[7]	0.60	[7]
98	Ethyl acetate	141-78-6	Lycopersicum esculentum Mill. (tomato fruit)	MX	2.29	[7,9]	-1.92	[7]	0.37	[9]

ID	**Compounds**	**CAS**	**Plant name**	**Tissue type**	**Log K_{ca}**	**Ref**	**Log K_{aw}**	**Ref**	**Log K_{cw}**	**Ref**
99	heptane	142-82-5	Lycopersicum esculentum Mill. (tomato fruit)	MX	2.51	[7]	-	-	-	-
100	heptane	142-82-5	Lycopersicum esculentum Mill. (tomato fruit)	MX	2.56	[8,9]	1.91	[8]	4.47	[9]
101	Monuron	150-68-5	*Capsicum* (pepper)	CM	9.30	[3,10]	-7.63	[3,10]	1.67	[10]
102	Monuron	150-68-5	Lycopersicum esculentum Mill. (tomato fruit)	CM	9.21	[3,10]	-7.63	[3,10]	1.58	[10]
103	Chlorotoluron	15545-48-9	*Capsicum* (pepper)	CM	9.80	[3,10]	-7.64	[3,10]	2.16	[10]
104	Chlorotoluron	15545-48-9	Lycopersicum esculentum Mill. (tomato fruit)	CM	9.73	[3,10]	-7.64	[3,10]	2.09	[10]
105	Atrazine	1912-24-9	*Capsicum* (pepper)	CM	9.29	[3,4]	-7.10	[3,4]	2.19	[4]
106	Atrazine	1912-24-9	*Capsicum* (pepper)	MX	9.30	[3,4]	-7.10	[3,4]	2.20	[4]
107	Atrazine	1912-24-9	Citrus	CM	9.25	[3,4]	-7.10	[3,4]	2.15	[4]
108	Atrazine	1912-24-9	Citrus	MX	9.27	[3,4]	-7.10	[3,4]	2.17	[4]
109	Atrazine	1912-24-9	Ficus (rubber plant)	CM	9.26	[3,4]	-7.10	[3,4]	2.16	[4]
110	Atrazine	1912-24-9	Ficus (rubber plant)	MX	9.25	[3,4]	-7.10	[3,4]	2.15	[4]
111	Atrazine	1912-24-9	Lycopersicum esculentum Mill. (tomato fruit)	CM	9.22	[3,4]	-7.10	[3,4]	2.12	[4]
112	Atrazine	1912-24-9	Lycopersicum esculentum Mill. (tomato fruit)	MX	9.23	[3,4]	-7.10	[3,4]	2.13	[4]
113	Atrazine	1912-24-9	Prunus laurocerasus L. (cherry laurel leaf)	CM	9.00	[3,4]	-7.10	[3,4]	1.90	[5]
114	Benzo[ghi]perylene	191-24-2	Euonymus japonicus	CM	13.99	[3,16]	-6.58	[3,16]	7.41	[16]
115	Perylene	198-55-0	*Capsicum* (pepper)	CM	12.29	[3,4]	-5.74	[3,4]	6.55	[4]
116	Perylene	198-55-0	*Capsicum* (pepper)	MX	12.32	[3,4]	-5.74	[3,4]	6.58	[4]
117	Perylene	198-55-0	Citrus	CM	12.19	[3,4]	-5.74	[3,4]	6.45	[4]
118	Perylene	198-55-0	Citrus	MX	12.33	[3,4]	-5.74	[3,4]	6.59	[4]
119	Perylene	198-55-0	Ficus (rubber plant)	CM	11.94	[3,4]	-5.74	[3,4]	6.20	[4]
120	Perylene	198-55-0	Ficus (rubber plant)	MX	12.32	[3,4]	-5.74	[3,4]	6.58	[4]

ID	**Compounds**	**CAS**	**Plant name**	**Tissue type**	**Log K_{ca}**	**Ref**	**Log K_{aw}**	**Ref**	**Log K_{cw}**	**Ref**
121	Perylene	198-55-0	Lycopersicum esculentum Mill. (tomato fruit)	CM	12.24	[3,4]	-5.74	[3,4]	6.50	[4]
122	Perylene	198-55-0	Lycopersicum esculentum Mill. (tomato fruit)	MX	12.23	[3,4]	-5.74	[3,4]	6.49	[4]
123	Diethyl suberate	2050-23-9	Madagascar jasmine	CM	7.13	[3,17]	-5.17	[3,17]	1.96	[17]
124	Decachlorobiphenyl	2051-24-3	Myriophyllum spicatum	Whole	6.15	[8,15]	-0.42	[8]	5.73	[15]
125	PCB 3	2051-62-9	Rhododendron L.	CM	5.60	[19]	-2.10	[19]	3.50	[19]
126	Fluoranthene	206-44-0	Euonymus japonicus	CM	9.33	[3,16]	-3.44	[3,16]	5.89	[16]
127	Cyanazine	21725-46-2	*Capsicum* (pepper)	CM	9.94	[3,20]	-8.14	[3,20]	1.80	[20]
128	Chrysene	218-01-9	Euonymus japonicus	CM	11.20	[3,16]	-4.79	[3,16]	6.41	[16]
129	Tetrachlorobiphenyl	26914-33-0	Myriophyllum spicatum	Whole	5.08	[8,15]	-1.38	[8]	3.70	[15]
130	PCB 118	31508-00-6	Rhododendron L.	CM	7.80	[19]	-2.40	[19]	5.40	[19]
131	Diuron	330-54-1	*Capsicum* (pepper)	CM	10.45	[3,10]	-7.97	[3,10]	2.48	[10]
132	Diuron	330-54-1	Lycopersicum esculentum Mill. (tomato fruit)	CM	10.42	[3,10]	-7.97	[3,10]	2.45	[10]
133	Isoproturon	34123-59-6	*Capsicum* (pepper)	CM	10.82	[3,10]	-9.00	[3,10]	1.82	[10]
134	Isoproturon	34123-59-6	Lycopersicum esculentum Mill. (tomato fruit)	CM	10.76	[3,10]	-9.00	[3,10]	1.76	[10]
135	Isoproturon	34123-59-6	Myriophyllum spicatum	whole	9.94	[3,10,21]	-9.00	[3,10]	0.94	[21]
136	PCB 138	35065-28-2	Rhododendron L.	CM	7.60	[19]	-1.90	[19]	5.70	[19]
137	PCB 180	35065-29-3	Rhododendron L.	CM	9.19	[8,19]	-3.39	[8]	5.80	[19]
138	PCB 52	35693-99-3	Rhododendron L.	CM	6.60	[19]	-2.00	[19]	4.60	[19]
139	PCB 101	37680-73-2	Rhododendron L.	CM	7.20	[19]	-2.10	[19]	5.10	[19]
140	bifenox	42576-02-3	*Capsicum* (pepper)	CM	9.80	[8,20]	-5.36	[8]	4.44	[20]
141	Benzo[a]pyrene	50-32-8	Euonymus japonicus	CM	12.55	[3,16]	-5.54	[3,16]	7.01	[16]
142	dibenz[a, h]anthracene	53-70-3	Euonymus japonicus	CM	12.79	[8,16]	-5.24	[8]	7.55	[16]
143	Carbontetrachloride	56-23-5	Lycopersicum esculentum Mill. (tomato fruit)	MX	2.43	[7]	-	-	-	-

ID	Compounds	CAS	Plant name	Tissue type	Log K_{ca}	Ref	Log K_{aw}	Ref	Log K_{cw}	Ref
144	Carbontetrachloride	56-23-5	Lycopersicum esculentum Mill. (tomato fruit)	MX	2.44	[8,9]	0.05	[8]	2.49	[9]
145	Benz[a]anthracene	56-55-3	Euonymus japonicus	CM	11.16	[3,16]	-4.59	[3,16]	6.57	[16]
146	2-Pentanol	6032-29-7	Lycopersicum esculentum Mill. (tomato fruit)	MX	3.68	[7,13]	-3.21	[7]	0.46	[13]
147	pentachlorobenzene	608-93-5	Myriophyllum spicatum	whole	4.68	[8,15]	-1.54	[8]	3.14	[15]
148	2-Hexanol	626-93-7	Lycopersicum esculentum Mill. (tomato fruit)	MX	4.08	[7,13]	-3.07	[7]	1.00	[7,13]
149	2-Hexanol	626-93-7	Lycopersicum esculentum Mill. (tomato fruit)	MX	4.08	[7,9]	-3.07	[7]	1.01	[9]
150	Carbaryl	63-25-2	Prunus laurocerasus L. (cherry laurel leaf)	CM	9.63	[3,22]	-7.46	[3,22]	2.17	[22]
151	Phenylurea	64-10-8	*Capsicum* (pepper)	CM	9.73	[3,20]	-8.86	[3,20]	0.87	[20]
152	Ethanol	64-17-5	Hedera helix L.	CM	2.74	[8,23]	-3.69	[8]	-0.95	[23]
153	Ethanol	64-17-5	Lycopersicum esculentum Mill. (tomato fruit)	MX	2.81	[7,13]	-3.67	[7]	-0.85	[13]
154	Ethanol	64-17-5	Lycopersicum esculentum Mill. (tomato fruit)	MX	2.81	[7,9]	-3.67	[7]	-0.86	[9]
155	Benzoic acid	65-85-0	*Capsicum* (pepper)	MX	6.68	[3,4]	-5.10	[3,4]	1.58	[4]
156	Benzoic acid	65-85-0	Chenopodium album	MX	6.86	[3,24]	-5.10	[3,24]	1.76	[24]
157	Benzoic acid	65-85-0	Ginkgo biloba L. (ginkgo leaf)	CM	6.82	[3,5]	-5.10	[3,5]	1.72	[5]
158	Benzoic acid	65-85-0	Hedera helix L.	CM	6.64	[3,4,23]	-5.10	[3,4]	1.54	[23]
159	Benzoic acid	65-85-0	Juglans regia L. (English walnut leaf)	CM	6.82	[3,5]	-5.10	[3,5]	1.72	[5]
160	Benzoic acid	65-85-0	Prunus laurocerasus L. (cherry laurel leaf)	CM	6.78	[3,5]	-5.10	[3,5]	1.68	[5]
161	Methanol	67-56-1	Citrus	CM	2.59	[3,4]	-3.74	[3,4]	-1.15	[4]
162	Methanol	67-56-1	Lycopersicum esculentum Mill. (tomato fruit)	MX	2.66	[7,13]	-3.71	[7]	-1.06	[13]
163	Methanol	67-56-1	Lycopersicum esculentum Mill. (tomato fruit)	MX	2.62	[7,9]	-3.71	[7]	-1.09	[9]

ID	Compounds	CAS	Plant name	Tissue type	Log K_{ca}	Ref	Log K_{aw}	Ref	Log K_{cw}	Ref
164	2-Propanol	67-63-0	Lycopersicum esculentum Mill. (tomato fruit)	MX	2.88	[7,13]	-3.49	[7]	-0.60	[13]
165	Acetone	67-64-1	Lycopersicum esculentum Mill. (tomato fruit)	MX	2.40	[7]	-2.38	[7]	0.04	[7]
166	Acetone	67-64-1	Lycopersicum esculentum Mill. (tomato fruit)	MX	1.99	[7,9]	-2.38	[7]	-0.39	[9]
167	Chloroform	67-66-3	Lycopersicum esculentum Mill. (tomato fruit)	MX	2.63	[7]	-0.92	[7]	1.73	[7]
168	Chloroform	67-66-3	Lycopersicum esculentum Mill. (tomato fruit)	MX	2.76	[7,9]	-0.92	[7]	1.84	[9]
169	Salicylic acid	69-72-7	Ginkgo biloba L. (ginkgo leaf)	CM	7.42	[3,5]	-5.39	[3,5]	2.03	[5]
170	Salicylic acid	69-72-7	Hedera helix L.	CM	7.09	[3,5,23]	-5.39	[3,5]	1.70	[23]
171	Salicylic acid	69-72-7	Juglans regia L. (English walnut leaf)	CM	7.37	[3,5]	-5.39	[3,5]	1.98	[5]
172	Salicylic acid	69-72-7	Prunus laurocerasus L. (cherry laurel leaf)	CM	7.48	[3,5]	-5.39	[3,5]	2.09	[5]
173	maltose	69-79-4	Hedera helix L.	CM	4.19	[8,23]	-3.69	[8]	0.50	[23]
174	PCB 28	7012-37-5	Rhododendron L.	CM	6.60	[19]	-1.90	[19]	4.70	[19]
175	1-Propanol	71-23-8	Lycopersicum esculentum Mill. (tomato fruit)	MX	3.24	[7,13]	-3.56	[7]	-0.32	[13]
176	1-Propanol	71-23-8	Lycopersicum esculentum Mill. (tomato fruit)	MX	3.26	[7,9]	-3.56	[7]	-0.30	[9]
177	1-Butanol	71-36-3	Lycopersicum esculentum Mill. (tomato fruit)	MX	3.70	[7,13]	-3.42	[7]	0.28	[13]
178	1-Butanol	71-36-3	Lycopersicum esculentum Mill. (tomato fruit)	MX	3.66	[7,9]	-3.42	[7]	0.24	[9]
179	1-Pentanol	71-41-0	Lycopersicum esculentum Mill. (tomato fruit)	MX	4.10	[7,13]	-3.28	[7]	0.81	[13]
180	1-Pentanol	71-41-0	Lycopersicum esculentum Mill. (tomato fruit)	MX	4.04	[7,9]	-3.28	[7]	0.76	[9]
181	Benzene	71-43-2	Lolium multiflorum Lam. (annual rye grass)	Whole	2.73	[25]	-1.38	[25]	1.35	[25]

ID	**Compounds**	**CAS**	**Plant name**	**Tissue type**	**Log K_{ca}**	**Ref**	**Log K_{aw}**	**Ref**	**Log K_{cw}**	**Ref**
182	Benzene	71-43-2	Lycopersicum esculentum Mill. (tomato fruit)	MX	2.63	[7]	-0.60	[7]	2.04	[7]
183	Benzene	71-43-2	Lycopersicum esculentum Mill. (tomato fruit)	MX	2.60	[7,9]	-0.60	[7]	2.00	[9]
184	1,1,1-Trichloroethane	71-55-6	Lycopersicum esculentum Mill. (tomato fruit)	MX	2.58	[7]	-0.18	[8]	-	-
185	1,1,1-Trichloroethane	71-55-6	Lycopersicum esculentum Mill. (tomato fruit)	MX	2.62	[8,9]	-0.18	[8]	2. [8]	[9]
186	Acetonitrile	75-05-8	Lycopersicum esculentum Mill. (tomato fruit)	MX	2.58	[7]	-2.92	[7]	-0.36	[7]
187	Acetonitrile	75-05-8	Lycopersicum esculentum Mill. (tomato fruit)	MX	2.65	[7,9]	-2.92	[7]	-0.27	[9]
188	Dichloromethane	75-09-2	Lycopersicum esculentum Mill. (tomato fruit)	MX	2.38	[7]	-3.96	[7]	1.41	[7]
189	Dichloromethane	75-09-2	Lycopersicum esculentum Mill. (tomato fruit)	MX	5.38	[7,9]	-3.96	[7]	1.42	[9]
190	1,1-dichloroethylene	75-35-4	Lycopersicum esculentum Mill. (tomato fruit)	MX	1.86	[7]	-	-	-	-
191	1,1-dichloroethylene	75-35-4	Lycopersicum esculentum Mill. (tomato fruit)	MX	2.01	[8,9]	0.03	[8]	2.04	[9]
192	2-methyl-2-propanol	75-65-0	Lycopersicum esculentum Mill. (tomato fruit)	MX	2.91	[7,13]	-3.30	[7]	-0.39	[13]
193	2-methyl-2-propanol	75-65-0	Lycopersicum esculentum Mill. (tomato fruit)	MX	2.93	[7,9]	-3.30	[7]	-0.37	[9]
194	2-Methyl-2-butanol	75-85-4	Lycopersicum esculentum Mill. (tomato fruit)	MX	3.36	[7,13]	-3.24	[7]	0.11	[13]
195	Trichloronitromethane	76-06-2	Lycopersicum esculentum Mill. (tomato fruit)	MX	3.28	[7]	-1.00	[7]	2.28	[7]
196	Trichloronitromethane	76-06-2	Lycopersicum esculentum Mill. (tomato fruit)	MX	3.13	[7,9]	-1.00	[7]	2.13	[9]
197	Paclobutrazol	76738-62-0	*Capsicum* (pepper)	CM	11.75	[26]	-9.39	[26]	2.36	[26]
198	Paclobutrazol	76738-62-0	Lycopersicum esculentum Mill. (tomato fruit)	CM	11.98	[26]	-9.39	[26]	2.59	[26]
199	Paclobutrazol	76738-62-0	Pyrus communis (pears)	CM	11.89	[26]	-9.39	[26]	2.50	[26]

ID	Compounds	CAS	Plant name	Tissue type	Log K_{ca}	Ref	Log K_{aw}	Ref	Log K_{cw}	Ref
200	Paclobutrazol	76738-62-0	Vanilla sp.	CM	11.02	[26]	-9.39	[26]	1.63	[26]
201	3-Methyl-3-pentanol	77-74-7	Lycopersicum esculentum Mill. (tomato fruit)	MX	3.69	[7,13]	-3.07	[7]	0.61	[13]
202	Isoprene	78-79-5	Lycopersicum esculentum Mill. (tomato fruit)	MX	1.59	[7]	0.50	[8]	-	-
203	Isoprene	78-79-5	Lycopersicum esculentum Mill. (tomato fruit)	MX	1.59	[8,9]	0.50	[8]	2.09	[9]
204	2-Methyl-1-propanol	78-83-1	Lycopersicum esculentum Mill. (tomato fruit)	MX	3.41	[7,13]	-3.29	[7]	0.11	[13]
205	1,2-Dichloropropane	78-87-5	Lycopersicum esculentum Mill. (tomato fruit)	MX	2.89	[7]	-1.13	[7]	1.76	[7]
206	1,2-Dichloropropane	78-87-5	Lycopersicum esculentum Mill. (tomato fruit)	MX	3.09	[7,9]	-1.13	[7]	1.96	[9]
207	2-Butanol	78-92-2	Lycopersicum esculentum Mill. (tomato fruit)	MX	3.29	[7,13]	-3.31	[7]	-0.02	[13]
208	2-Butanol	78-92-2	Lycopersicum esculentum Mill. (tomato fruit)	MX	3.22	[7,9]	-3.31	[7]	-0.09	[9]
209	2-Butanone	78-93-3	Lycopersicum esculentum Mill. (tomato fruit)	MX	2.56	[7]	-2.59	[7]	-0.04	[7]
210	2-Butanone	78-93-3	Lycopersicum esculentum Mill. (tomato fruit)	MX	2.43	[7,9]	-2.59	[7]	-0.16	[9]
211	Trichloroethylene	79-01-6	Lycopersicum esculentum Mill. (tomato fruit)	MX	2.88	[7]	-0.40	[8]	-	-
212	Trichloroethylene	79-01-6	Lycopersicum esculentum Mill. (tomato fruit)	MX	2.96	[8,9]	-0.40	[8]	2.56	[9]
213	Acenaphthene	83-32-9	Euonymus japonicus	CM	6.58	[3,16]	-2.31	[3,16]	4.27	[16]
214	Phenanthrene	85-01-8	*Capsicum* (pepper)	MX	7.66	[3,12]	-2.80	[3,12]	4.86	[12]
215	Phenanthrene	85-01-8	Euonymus japonicus	CM	7.99	[3,16]	-2.80	[3,16]	5.19	[16]
216	Phenanthrene	85-01-8	Lolium multiflorum Lam. (annual rye grass)	Whole	6.46	[3,16,25]	-2.80	[3,16]	3.66	[25]
217	Phenanthrene	85-01-8	Malus domestica (apple)	MX	7.56	[3,27]	-2.80	[3,27]	4.76	[27]
218	Phenanthrene	85-01-8	Pinus	CM	6.62	[3,16,18]	-2.80	[3,16]	3.82	[18]

ID	**Compounds**	**CAS**	**Plant name**	**Tissue type**	**Log K_{ca}**	**Ref**	**Log K_{aw}**	**Ref**	**Log K_{cw}**	**Ref**
219	Phenanthrene	85-01-8	Solanum lycopersicum (tomato fruit)	MX	7.54	[3,27]	-2.80	[3,27]	4.74	[27]
220	Phenanthrene	85-01-8	Solanum tuberosum	MX	7.10	[3,27]	-2.80	[3,27]	4.30	[27]
221	Phenanthrene	85-01-8	Vitis heyneana Roem. Et Schult	MX	7.39	[3,27]	-2.80	[3,27]	4.59	[27]
222	Pentachlorophenol	87-86-5	*Capsicum* (pepper)	CM	8.32	[3,4]	-3.66	[3,4]	4.66	[4]
223	Pentachlorophenol	87-86-5	*Capsicum* (pepper)	MX	8.38	[3,4]	-3.66	[3,4]	4.72	[4]
224	Pentachlorophenol	87-86-5	Citrus	CM	8.08	[3,4]	-3.66	[3,4]	4.42	[4]
225	Pentachlorophenol	87-86-5	Citrus	MX	8.12	[3,4]	-3.66	[3,4]	4.46	[4]
226	Pentachlorophenol	87-86-5	Ficus (rubber plant)	CM	8.21	[3,4]	-3.66	[3,4]	4.55	[4]
227	Pentachlorophenol	87-86-5	Ficus (rubber plant)	MX	8.26	[3,4]	-3.66	[3,4]	4.60	[4]
228	Pentachlorophenol	87-86-5	Lycopersicum esculentum Mill. (tomato fruit)	CM	8.23	[3,4]	-3.66	[3,4]	4.57	[4]
229	Pentachlorophenol	87-86-5	Lycopersicum esculentum Mill. (tomato fruit)	MX	8.36	[3,4]	-3.66	[3,4]	4.70	[4]
230	2,4,6-Trichlorophenol	88-06-2	*Capsicum* (pepper)	CM	6.92	[8,11]	-3.77	[8]	3.15	[11]
231	2,4,6-Trichlorophenol	88-06-2	Malus domestica (apple)	CM	6.67	[8,11]	-3.77	[8]	2.90	[11]
232	2,4,6-Trichlorophenol	88-06-2	Solanum lycopersicum (tomato fruit)	CM	6.93	[8,11]	-3.77	[8]	3.16	[11]
233	2,4,6-Trichlorophenol	88-06-2	Solanum tuberosum	CM	6.17	[8,11]	-3.77	[8]	2.40	[11]
234	2-Nitrophenol	88-75-5	*Capsicum* (pepper)	CM	5.36	[3,4]	-3.44	[3,4]	1.92	[4]
235	2-Nitrophenol	88-75-5	*Capsicum* (pepper)	MX	5.48	[3,4]	-3.44	[3,4]	2.04	[4]
236	2-Nitrophenol	88-75-5	Ficus (rubber plant)	CM	5.28	[3,4]	-3.44	[3,4]	1.84	[4]
237	2-Nitrophenol	88-75-5	Ficus (rubber plant)	MX	5.43	[3,4]	-3.44	[3,4]	1.99	[4]
238	2-Nitrophenol	88-75-5	Lycopersicum esculentum Mill. (tomato fruit)	CM	5.27	[3,4]	-3.44	[3,4]	1.83	[4]
239	2-Nitrophenol	88-75-5	Lycopersicum esculentum Mill. (tomato fruit)	MX	5.43	[3,4]	-3.44	[3,4]	1.99	[4]
240	1-Naphthalenol	90-15-3	*Capsicum* (pepper)	MX	8.80	[3,28]	-5.87	[3,28]	2.93	[28]

ID	**Compounds**	**CAS**	**Plant name**	**Tissue type**	**Log K_{ca}**	**Ref**	**Log K_{aw}**	**Ref**	**Log K_{cw}**	**Ref**
241	1-Naphthalenol	90-15-3	*Capsicum* (pepper)	MX	8.88	[3,12]	-5.87	[3,12]	3.01	[12]
242	1-Naphthalenol	90-15-3	Malus domestica (apple)	MX	8.91	[3,28]	-5.87	[3,28]	3.04	[28]
243	1-Naphthalenol	90-15-3	Solanum lycopersicum (tomato fruit)	MX	8.78	[3,28]	-5.87	[3,28]	2.91	[28]
244	Naphthalene	91-20-3	*Capsicum* (pepper)	MX	5.10	[3,28]	-1.73	[3,28]	3.37	[28]
245	Naphthalene	91-20-3	*Capsicum* (pepper)	MX	5.12	[3,12]	-1.73	[3,12]	3.39	[12]
246	Naphthalene	91-20-3	Malus domestica (apple)	MX	5.15	[3,28]	-1.73	[3,28]	3.42	[28]
247	Naphthalene	91-20-3	Solanum lycopersicum (tomato fruit)	MX	5.11	[3,28]	-1.73	[3,28]	3.38	[28]
248	o-Xylene	95-47-6	Lycopersicum esculentum Mill. (tomato fruit)	MX	3.56	[7]	-0.74	[7]	2.81	[7]
249	o-Xylene	95-47-6	Lycopersicum esculentum Mill. (tomato fruit)	MX	3.64	[7,9]	-0.74	[7]	2.90	[9]
250	1,2-Dichlorobenzene	95-50-1	Festuca rubra L. (red fescu)	Whole	4.05	[3,25]	-1.64	[3,25]	2.41	[25]
251	1,2-Dichlorobenzene	95-50-1	Lolium arundinaceium (tall fescu)	Whole	4.06	[3,25]	-1.64	[3,25]	2.42	[25]
252	1,2-Dichlorobenzene	95-50-1	Lolium multiflorum Lam. (annual rye grass)	Whole	4.06	[3,25]	-1.65	[3,25]	2.41	[25]
253	1,2-Dichlorobenzene	95-50-1	Spinacia oleracea (spinach)	Whole	3.96	[3,25]	-1.64	[3,25]	2.32	[25]
254	1,2,4,5-Tetrachlorobenzene	95-94-3	Myriophyllum spicatum	Whole	3.63	[8,15]	-1.39	[8]	2.24	[15]
255	cloquintocet-mexyl	99607-70-2	Hedera helix L.	CM	12.21	[8,23]	-7.48	[8]	4.73	[23]

Table S8. Values of log K_{ca} for Compounds in dataset (II).

ID	Compounds	CAS	SMILES	Log K_{ca}
1	4-Nitrophenol	100-02-7	O=[N+]([O-])c1ccc(O)cc1	9.41
2	4-Nitroanisole	100-17-4	COc1ccc([N+](=O)[O-])cc1	6.01
3	Ethylbenzene	100-41-4	CCc1ccccc1	3.36
4	Styrene	100-42-5	C=Cc1ccccc1	3.68
5	Fenuron	101-42-8	CN(C)C(=O)Nc1ccccc1	9.06
6	4-Chlorophenol	106-48-9	Oc1ccc(Cl)cc1	6.81
7	Epichlorohydrin	106-89-8	ClCC1CO1	3.34
8	1,2-Dibromoethane	106-93-4	BrCCBr	3.36
9	allyl chloride	107-05-1	C=CCCl	2.35
10	1,2-Dichloroethane	107-06-2	ClCCCl	2.75
11	Acrylonitrile	107-13-1	C=CC#N	2.15
12	1-Nitropropane	108-03-2	CCC[N+](=O)[O-]	3.48
13	4-Methyl-2-pentanone	108-10-1	CC(=O)CC(C)C	2.95
14	Toluene	108-88-3	Cc1ccccc1	3.10
15	Chlorobenzene	108-90-7	Clc1ccccc1	3.52
16	Cyclohexanone	108-94-1	O=C1CCCCC1	3.84
17	Phenol	108-95-2	Oc1ccccc1	6.57
18	propyl acetate	109-60-4	CCCOC(C)=O	2.80
19	Tetrahydrofuran	109-99-9	C1CCOC1	3.00
20	Cyclohexane	110-82-7	C1CCCCC1	2.29
21	Pyridine	110-86-1	c1ccncc1	3.80
22	1-Hexanol	111-27-3	CCCCCCO	4.48
23	bis(2-Ethylhexyl) phthalate	117-81-7	CCCCC(CC)COC(=O)c1ccccc1C(=O)OC C(CC)CCCC	12.11
24	Hexachlorobenzene	118-74-1	Clc1c(Cl)c(Cl)c(Cl)c(Cl)c1Cl	6.71
25	TNT	118-96-7	Cc1c([N+](=O)[O-])cc([N+](=O)[O-])cc1 [N+](=O)[O-]	8.59
26	2,4-Dinitroanisole	119-27-7	COc1ccc([N+](=O)[O-])cc1[N+](=O)[O-]	7.85
27	Anthracene	120-12-7	c1ccc2cc3ccccc3cc2c1	8.23
28	2,4-Dichlorophenol	120-83-2	Oc1ccc(Cl)cc1Cl	7.61
29	2,4-Dinitrotoluene	121-14-2	Cc1ccc([N+](=O)[O-])cc1[N+](=O)[O-]	7.05
30	Butyl acetate	123-86-4	CCCCOC(C)=O	3.22
31	1,4-Dioxane	123-91-1	C1COCCO1	3.14
32	Tributyl phosphate	126-73-8	CCCCOP(=O)(OCCCC)OCCCC	7.36
33	Tetrachloroethylene	127-18-4	ClC(Cl)=C(Cl)Cl	2.98
34	Pyrene	129-00-0	c1cc2ccc3cccc4ccc(c1)c2c34	8.79
35	PCB 4	13029-08-8	Clc1ccccc1-c1ccccc1Cl	5.00
36	Limonene	138-86-3	C=C(C)C1CC=C(C)CC1	3.95
37	Ethyl acetate	141-78-6	CCOC(C)=O	2.41
38	heptane	142-82-5	CCCCCCC	2.53

ID	Compounds	CAS	SMILES	Log K_{ca}
39	Monuron	150-68-5	CN(C)C(=O)Nc1ccc(Cl)cc1	9.26
40	Chlorotoluron	15545-48-9	Cc1ccc(NC(=O)N(C)C)cc1Cl	9.77
41	Atrazine	1912-24-9	CCNc1nc(Cl)nc(NC(C)C)n1	9.23
42	Benzo[ghi]perylene	191-24-2	c1cc2ccc3ccc4ccc5cccc6c(c1)c2c3c4c56	13.99
43	Perylene	198-55-0	c1cc2cccc3c4cccc5cccc(c(c1)c23)c54	12.23
44	Diethyl suberate	2050-23-9	CCOC(=O)CCCCCCC(=O)OCC	7.13
45	Decachlorobiphenyl	2051-24-3	Clc1c(Cl)c(Cl)c(-c2c(Cl)c(Cl)c(Cl)c(Cl)c2Cl)c(Cl)c1Cl	6.15
46	PCB 3	2051-62-9	Clc1ccc(-c2ccccc2)cc1	5.60
47	Fluoranthene	206-44-0	c1ccc2c(c1)-c1cccc3cccc-2c13	9.33
48	Cyanazine	21725-46-2	CCNc1nc(Cl)nc(NC(C)(C)C#N)n1	9.94
49	Chrysene	218-01-9	c1ccc2c(c1)ccc1c3ccccc3ccc21	11.20
50	Tetrachlorobiphenyl	26914-33-0	Clc1cc(-c2ccccc2)c(Cl)c(Cl)c1Cl	5.08
51	PCB 118	31508-00-6	Clc1ccc(-c2cc(Cl)c(Cl)cc2Cl)cc1Cl	7.80
52	Diuron	330-54-1	CN(C)C(=O)Nc1ccc(Cl)c(Cl)c1	10.44
53	Isoproturon	34123-59-6	CC(C)c1ccc(NC(=O)N(C)C)cc1	10.51
54	PCB 138	35065-28-2	Clc1cc(Cl)c(-c2ccc(Cl)c(Cl)c2Cl)cc1Cl	7.60
55	PCB 180	35065-29-3	Clc1cc(Cl)c(-c2cc(Cl)c(Cl)c(Cl)c2Cl)cc1Cl	9.19
56	PCB 52	35693-99-3	Clc1ccc(Cl)c(-c2cc(Cl)ccc2Cl)c1	6.60
57	PCB 101	37680-73-2	Clc1ccc(Cl)c(-c2cc(Cl)c(Cl)cc2Cl)c1	7.20
58	bifenox	42576-02-3	COC(=O)c1cc(Oc2ccc(Cl)cc2Cl)ccc1[N+](=O)[O-]	9.80
59	Benzo[a]pyrene	50-32-8	c1ccc2c(c1)cc1ccc3cccc4ccc2c1c34	12.55
60	dibenz[a, h]anthracene	53-70-3	c1ccc2c(c1)ccc1cc3c(ccc4ccccc43)cc12	12.79
61	Carbontetrachloride	56-23-5	ClC(Cl)(Cl)Cl	2.43
62	Benz[a]anthracene	56-55-3	c1ccc2cc3c(ccc4ccccc43)cc2c1	11.16
63	2-Pentanol	6032-29-7	CCCC(C)O	3.68
64	pentachlorobenzene	608-93-5	Clc1cc(Cl)c(Cl)c(Cl)c1Cl	4.68
65	2-Hexanol	626-93-7	CCCCC(C)O	4.08
66	Carbaryl	63-25-2	CNC(=O)Oc1cccc2ccccc12	9.63
67	Phenylurea	64-10-8	NC(=O)Nc1ccccc1	9.73
68	Ethanol	64-17-5	CCO	2.79
69	Benzoic acid	65-85-0	O=C(O)c1ccccc1	6.77
70	Methanol	67-56-1	CO	2.62
71	2-Propanol	67-63-0	CC(C)O	2.88
72	Acetone	67-64-1	CC(C)=O	2.19
73	Chloroform	67-66-3	ClC(Cl)Cl	2.70
74	Salicylic acid	69-72-7	O=C(O)c1ccccc1O	7.34
75	maltose	69-79-4	O=C[C@H](O)[C@@H](O)[C@H](O[C@H]1O[C@H](CO)[C@@H](O)[C@H](O)[C@H]1O)[C@H](O)CO	4.19

ID	Compounds	CAS	SMILES	Log K_{ca}
76	PCB 28	7012-37-5	Clc1ccc(-c2ccc(Cl)cc2Cl)cc1	6.60
77	1-Propanol	71-23-8	CCCO	3.25
78	1-Butanol	71-36-3	CCCCO	3.68
79	1-Pentanol	71-41-0	CCCCCO	4.07
80	Benzene	71-43-2	c1ccccc1	2.65
81	1,1,1-Trichloroethane	71-55-6	CC(Cl)(Cl)Cl	2.60
82	Acetonitrile	75-05-8	CC#N	2.62
83	Dichloromethane	75-09-2	ClCCl	3.88
84	1,1-dichloroethylene	75-35-4	C=C(Cl)Cl	1.94
85	2-methyl-2-propanol	75-65-0	CC(C)(C)O	2.92
86	2-Methyl-2-butanol	75-85-4	CCC(C)(C)O	3.36
87	Trichloronitromethane	76-06-2	O=[N+]([O-])C(Cl)(Cl)Cl	3.21
88	Paclobutrazol	76738-62-0	CC(C)(C)[C@H](O)[C@H](Cc1ccc(Cl)cc1)n1cncn1	11.66
89	3-Methyl-3-pentanol	77-74-7	CCC(C)(O)CC	3.69
90	Isoprene	78-79-5	C=CC(=C)C	1.59
91	2-Methyl-1-propanol	78-83-1	CC(C)CO	3.41
92	1,2-Dichloropropane	78-87-5	CC(Cl)CCl	2.99
93	2-Butanol	78-92-2	CCC(C)O	3.26
94	2-Butanone	78-93-3	CCC(C)=O	2.49
95	Trichloroethylene	79-01-6	ClC=C(Cl)Cl	2.92
96	Acenaphthene	83-32-9	c1cc2c3c(cccc3c1)CC2	6.58
97	Phenanthrene	85-01-8	c1ccc2c(c1)ccc1ccccc12	7.29
98	Pentachlorophenol	87-86-5	Oc1c(Cl)c(Cl)c(Cl)c(Cl)c1Cl	8.25
99	2,4,6-Trichlorophenol	88-06-2	Oc1c(Cl)cc(Cl)cc1Cl	6.67
100	2-Nitrophenol	88-75-5	O=[N+]([O-])c1ccccc1O	5.38
101	1-Naphthalenol	90-15-3	Oc1cccc2ccccc12	8.84
102	Naphthalene	91-20-3	c1ccc2ccccc2c1	5.12
103	o-Xylene	95-47-6	Cc1ccccc1C	3.60
104	1,2-Dichlorobenzene	95-50-1	Clc1ccccc1Cl	4.03
105	1,2,4,5-Tetrachlorobenzene	95-94-3	Clc1cc(Cl)c(Cl)cc1Cl	3.63
106	cloquintocet-mexyl	99607-70-2	CCCCCC(C)OC(=O)COc1ccc(Cl)c2cccnc12	12.21

Notes: The log K_{ca} values of compounds in dataset (II) are calculated by averaging the log K_{ca} values corresponding to different plant species or tissue types of the same compound in dataset (I).

Figures

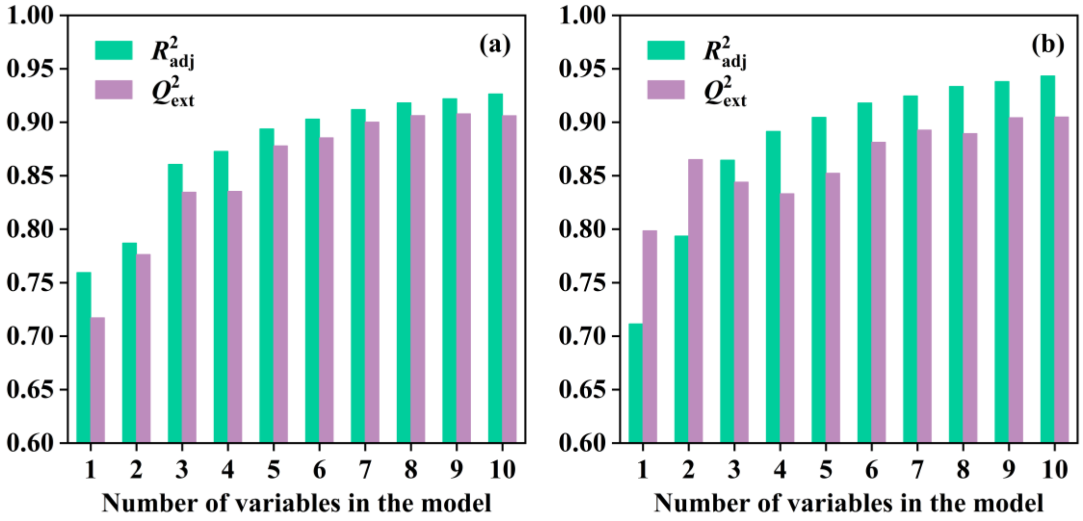

Figure S1. The bar and line plots show the R^2_{adj} and Q^2_{ext} of the QSPR models. (a) Dataset (I); (b) Dataset (II).

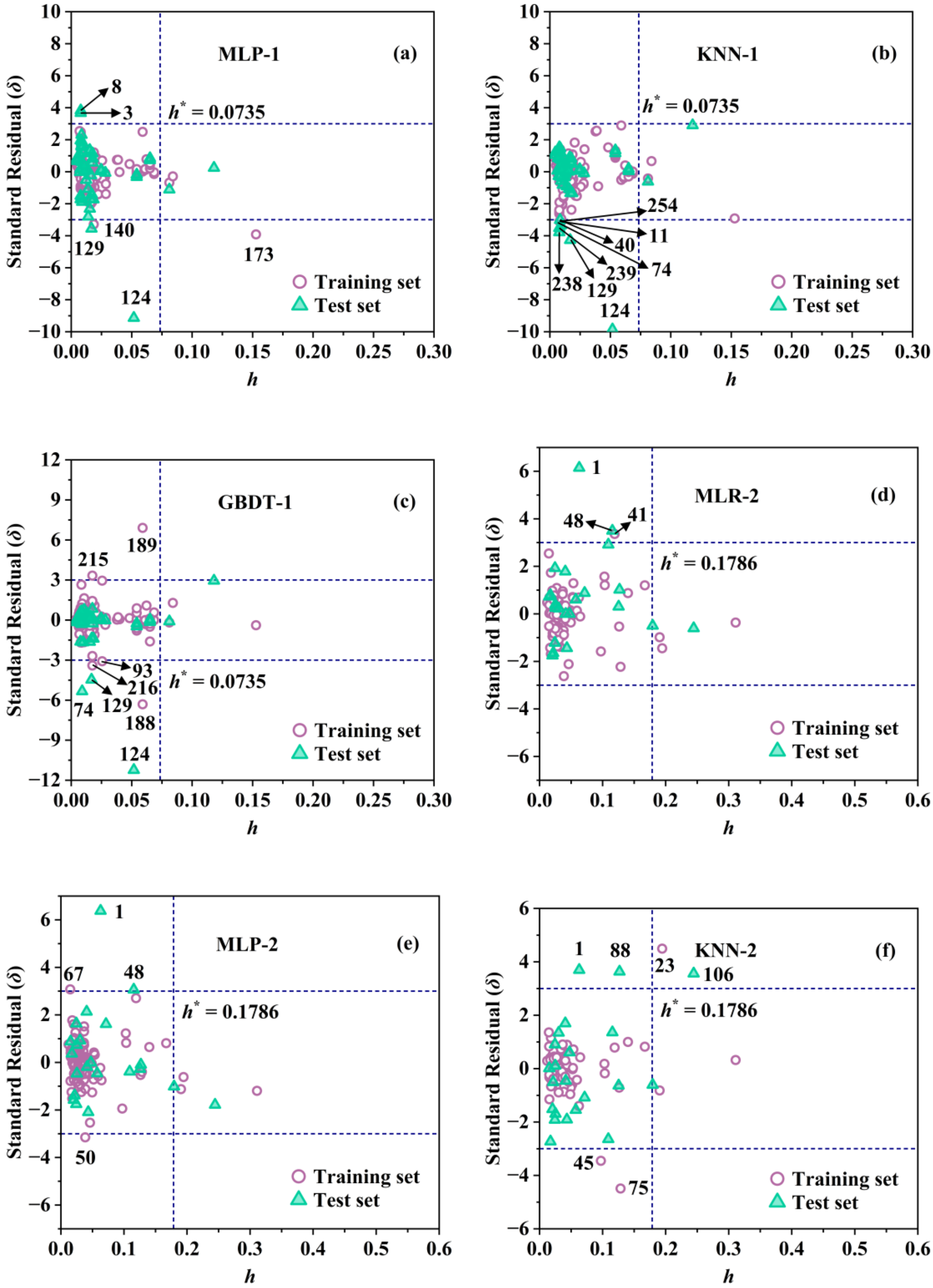

Figure S2. Application domain characterized by Williams plots: the MLP-1 (a), KNN-1 (b), GBDT-1 (c), MLR-2 (d), MLP-2 (e) and KNN-2 (f) models for log K_{ca}.

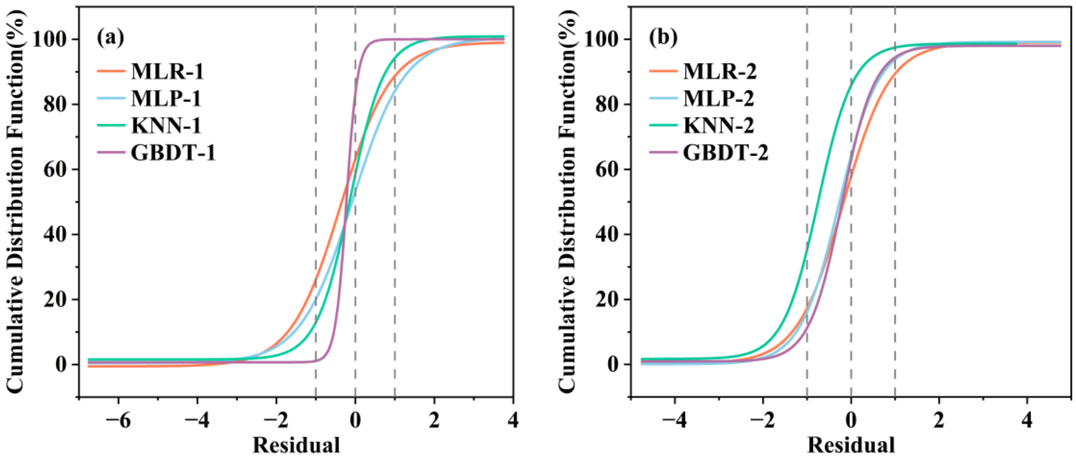

Figure S3. Cumulative distributions of residuals between the observed and predicted log K_{ca}. (a) Dataset (I); (b) Dataset (II).

Reference

[1] S.M. Lundberg, G. Erion, H. Chen, A. DeGrave, J.M. Prutkin, B. Nair, R. Katz, J. Himmelfarb, N. Bansal, S.-I. Lee, From local explanations to global understanding with explainable AI for trees, Nat. Mach. Intell. 2 (2020) 56–67. https://doi.org/10.1038/s42256-019-0138-9.

[2] L.S. Shapley, Contributions to the Theory of Games (AM-28), Volume II, in: H.W. Kuhn, A.W. Tucker (Eds.), Princeton University Press, 1953: pp. 307–318. https://doi.org/10.1515/9781400881970-018.

[3] S. Eddula, A. Xu, C. Jiang, J. Huang, P. Tirumala, G. Liu, W.E. Acree, M.H. Abraham, Abraham solvation parameter model: updated correlations for describing solute partitioning into plant cuticles from water and from air, Phys. Chem. Liq. 59 (2021) 716–732. https://doi.org/10.1080/00319104.2020.1808659.

[4] A. Sabljic, H. Guesten, J. Schoenherr, M. Riederer, Modeling plant uptake of airborne organic chemicals. 1. Plant cuticle/water partitioning and molecular connectivity, Environ. Sci. Technol. 24 (1990) 1321–1326. https://doi.org/10.1021/es00079a004.

[5] T. Kirsch, F. Kaffarnik, M. Riederer, L. Schreiber, Cuticular permeability of the three tree species Prunus Iaurocerasus L., Ginkgo biloba L. and Juglans regia L.: comparative investigation of the transport properties of intact leaves, isolated cuticles and reconstituted cuticular waxes, J. Exp. Bot. 48 (1997) 1035–1045. https://doi.org/10.1093/jxb/48.5.1035.

[6] T.L. Torralba−Sanchez, Y. Liang, D.M. Di Toro, Estimating Grass–Soil Bioconcentration of Munitions Compounds from Molecular Structure, Environ. Sci. Technol. 51 (2017) 11205–11214. https://doi.org/10.1021/acs.est.7b02572.

[7] B. Welke, K. Ettlinger, M. Riederer, Sorption of Volatile Organic Chemicals in Plant Surfaces, Environ. Sci. Technol. 32 (1998) 1099–1104. https://doi.org/10.1021/es970763v.

[8] O. US EPA, EPI Suite^{TM}-Estimation Program Interface, (2015). https://www.epa.gov/tsca-screening-tools/epi-suitetm-estimation-program-interface (accessed September 18, 2023).

[9] J.A. Platts, M.H. Abraham, Partition of Volatile Organic Compounds from Air and from Water into Plant Cuticular Matrix: An LFER Analysis, Environ. Sci. Technol. 34 (2000) 318–323. https://doi.org/10.1021/es9906195.

[10] C. Evelyne, C. André, T. Georges, T. Michel, Quantitative relationships between structure and penetration of phenylurea herbicides through isolated plant cuticles, Chemosphere. 24 (1992) 189–200. https://doi.org/10.1016/0045-6535(92)90392-5.

[11] Y. Li, Y. Deng, B. Chen, Sorption of chlorophenols onto fruit cuticles and potato periderm, J. Environ. Sci. 24 (2012) 675–681. https://doi.org/10.1016/S1001-0742(11)60891-7.

[12] B. Chen, E.J. Johnson, B. Chefetz, L. Zhu, B. Xing, Sorption of Polar and Nonpolar Aromatic Organic Contaminants by Plant Cuticular Materials: Role of Polarity and Accessibility, Environ. Sci. Technol. 39 (2005) 6138–6146. https://doi.org/10.1021/es050622q.

[13] S. Merk, M. Riederer, Sorption of volatile C1 to C6 alkanols in plant cuticles, J. Exp. Bot. 48 (1997) 1095–1104. https://doi.org/10.1093/jxb/48.5.1095.

[14] E. Bacci, D. Calamari, C. Gaggi, M. Vighi, Bioconcentration of organic chemical vapors in plant leaves: experimental measurements and correlation, Environ. Sci. Technol. 24 (1990) 885–889. https://doi.org/10.1021/es00076a015.

[15] F.A.P.C. Gobas, E.J. McNeil, L. Lovett-Doust, G.D. Haffner, Bioconcentration of chlorinated aromatic hydrocarbons in aquatic macrophytes, Environ. Sci. Technol. 25 (1991) 924–929. https://doi.org/10.1021/es00017a015.
[16] S.-J. Kim, H. Lee, J.-H. Kwon, Measurement of partition coefficients for selected polycyclic aromatic hydrocarbons between isolated plant cuticles and water, Sci. Total Environ. 494–495 (2014) 113–118. https://doi.org/10.1016/j.scitotenv.2014.06.119.
[17] T. Shi, J. Schönherr, L. Schreiber, Accelerators Increase Permeability of Cuticles for the Lipophilic Solutes Metribuzin and Iprovalicarb but Not for Hydrophilic Methyl Glucose, J. Agric. Food Chem. 53 (2005) 2609–2615. https://doi.org/10.1021/jf048242w.
[18] Y. Li, B. Chen, L. Zhu, Single-solute and bi-solute sorption of phenanthrene and pyrene onto pine needle cuticular fractions, Environ. Pollut. 158 (2010) 2478–2484. https://doi.org/10.1016/j.envpol.2010.03.021.
[19] D.J. Bolinius, M. MacLeod, M.S. McLachlan, P. Mayer, A. Jahnke, A passive dosing method to determine fugacity capacities and partitioning properties of leaves, Environ. Sci. Process. Impacts. 18 (2016) 1325–1332. https://doi.org/10.1039/C6EM00423G.
[20] P. Baur, H. Marzouk, J. Schönherr, B.T. Grayson, Partition Coefficients of Active Ingredients between Plant Cuticle and Adjuvants As Related to Rates of Foliar Uptake, J. Agric. Food Chem. 45 (1997) 3659–3665. https://doi.org/10.1021/jf970233i.
[21] S. Heine, W. Schmitt, A. Schäffer, G. Görlitz, H. Buresová, G. Arts, T.G. Preuss, Mechanistic modelling of toxicokinetic processes within Myriophyllum spicatum, Chemosphere. 120 (2015) 292–298. https://doi.org/10.1016/j.chemosphere.2014.07.065.
[22] C. Ballmann, S. De Oliveira, A. Gutenberger, F. Waßmann, L. Schreiber, A radioactive assay allowing the quantitative measurement of cuticular permeability of intact Arabidopsis thaliana leaves, Planta. 234 (2011) 9–20. https://doi.org/10.1007/s00425-011-1381-4.
[23] C. Popp, M. Burghardt, A. Friedmann, M. Riederer, Characterization of hydrophilic and lipophilic pathways of Hedera helix L. cuticular membranes: permeation of water and uncharged organic compounds, J. Exp. Bot. 56 (2005) 2797–2806. https://doi.org/10.1093/jxb/eri272.
[24] M. Burghardt, A. Friedmann, L. Schreiber, M. Riederer, Modelling the effects of alcohol ethoxylates on diffusion of pesticides in the cuticular wax of Chenopodium album leaves, Pest Manag. Sci. 62 (2006) 137–147. https://doi.org/10.1002/ps.1139.
[25] J.P. Barbour, J.A. Smith, C.T. Chiou, Sorption of Aromatic Organic Pollutants to Grasses from Water, Environ. Sci. Technol. 39 (2005) 8369–8373. https://doi.org/10.1021/es0504946.
[26] A. Chamel, B. Gambonnet, L. Arnaud, M. Alfi, Foliar absorption of [14C]paclobutrazol : study of cuticular sorption and penetration using isolated cuticles, Plant Physiol. Biochem. (1991). https://www.semanticscholar.org/paper/Foliar-absorption-of-%5B14C%5Dpaclobutrazol-%3A-study-of-Chamel-Gambonnet/7340009c8461d71a5e570145551c67f87aaff207 (accessed September 18, 2023).
[27] Y. Li, B. Chen, Phenanthrene Sorption by Fruit Cuticles and Potato Periderm with Different Compositional Characteristics, J. Agric. Food Chem. 57 (2009) 637–644.

https://doi.org/10.1021/jf802719h.
[28] B. Chen, Y. Li, Y. Guo, L. Zhu, J.L. Schnoor, Role of the Extractable Lipids and Polymeric Lipids in Sorption of Organic Contaminants onto Plant Cuticles, Environ. Sci. Technol. 42 (2008) 1517–1523. https://doi.org/10.1021/es7023725.
